# Phylogenomic characterisation of a novel corynebacterial species pathogenic to animals

**DOI:** 10.1007/s10482-020-01430-5

**Published:** 2020-06-04

**Authors:** Jens Möller, Luca Musella, Vyacheslav Melnikov, Walter Geißdörfer, Andreas Burkovski, Vartul Sangal

**Affiliations:** 1 Professur für Mikrobiologie, Friedrich-Alexander-Universität Erlangen-Nürnberg, Erlangen, Germany; 2 Gabrichevsky Research Institute for Epidemiology and Microbiology, Moscow, Russia; 3 Institute of Clinical Microbiology, Immunology and Hygiene, Universitätsklinikum Erlangen, Friedrich-Alexander-Universität Erlangen-Nürnberg, Erlangen, Germany; 4 Faculty of Health and Life Sciences, Northumbria University, Newcastle upon Tyne, UK

**Keywords:** *Corynebacterium ulcerans*, Diphtheria, Non-toxigenic, NTTB, Toxigenic, Virulence, Zoonotic

## Abstract

The genus *Corynebacterium* includes species of biotechnological, medical and veterinary importance. An atypical *C. ulcerans* strain, W25, was recently isolated from a case of necrotizing lymphadenitis in a wild boar. In this study, we have analysed the genome sequence of this strain and compared the phenotypic and virulence properties with other corynebacterial pathogens. Phylogenomic analyses revealed that strain W25 belongs to a novel species along with PO100/5 and KL1196. The latter strains were isolated from a pig and a roe deer, respectively; hence, this species appears to be associated to animals. The isolate W25 is likely a non-toxigenic *tox* gene bearing strain and may have compromised abilities to adhere to pharyngeal and laryngeal epithelial cells due to potential loss of the gene functions in *spaBC* and *spaDEF* pilus gene clusters. A number of corynebacterial virulence genes are present including *pld* encoding phospholipase D. Therefore, this strain may be able to cause severe invasive infections in animals and zoonotic infections in humans.

**Electronic supplementary material:**

The online version of this article (10.1007/s10482-020-01430-5) contains supplementary material, which is available to authorized users.

## Introduction

*Corynebacterium* is a diverse genus that includes species of biotechnological, medical and veterinary importance (Bernard and Funke 2015). One of the corynebacterial species, *Corynebacterium ulcerans,* is an important zoonotic pathogen often acquired from canine pets and causes diphtheria-like infections in humans (Hacker et al. 2016; Mattos-Guaraldi et al. 2014). *C. ulcerans* has also been isolated from other animals including camels, cattle, cats, goats, ground squirrels, monkeys, pigs, otters, whales, etc. [Reviewed by Hacker et al. (2016)]. A human-to-human transmission of *C. ulcerans* has also been reported (Konrad et al. 2015).

The key virulence factor among *C. ulcerans* strains is the *tox* gene (Sangal and Hoskisson 2014; Sangal et al. 2014; Subedi et al. 2018); which is borne by a corynephage (Sekizuka et al. 2012) or pathogenicity island (Meinel et al. 2014). However, non-toxigenic *tox* gene bearing (NTTB) *C. ulcerans* strains, where *tox* gene is a pseudogene, are also common (Dias et al. 2011; Eisenberg et al. 2014; Fuursted et al. 2015; Wagner et al. 2011; Zakikhany et al. 2014). Other known virulence-associated genes in *C. ulcerans* include *pld* (phospholipase D), *nanH* (neuraminidase H), *cp40* (corynebacterial protease), *vsp1* and *vsp2* (venom serine protease) and *rbp* (Sangal et al. 2014; Sekizuka et al. 2012; Subedi et al. 2018; Trost et al. 2011). The *rbp* gene encodes a ribosomal-binding protein that is similar to Shiga-like toxin and is only reported in *C. ulcerans* strain 809 (Subedi et al. 2018; Trost et al. 2011). Interestingly, an Rbp homolog was found in *C. diphtheriae* HC04 (Weerasekera et al. 2019).

Two Spa gene clusters, *spaDEF* and *spaBC*, have been reported among *C. ulcerans* strains (Subedi et al. 2018; Trost et al. 2011). Pilus gene clusters encode surface pili that play a key role in adhesion and invasion to the host cells (Broadway et al. 2013; Reardon-Robinson and Ton-That 2014). A variation in the numbers of pilus gene clusters and gain or loss of gene function was found to correlate with differences in the severity of infection by *Corynebacterium diphtheriae*, another important human pathogen closely related to *C. ulcerans* (Grosse-Kock et al. 2017; Ott et al. 2010; Sangal et al. 2015).

We have recently isolated an atypical *C. ulcerans* strain, W25, associated with necrotizing lymphadenitis in a wild boar and published the genome sequence (Busch et al. 2019). While the size of the genome is consistent with other *C. ulcerans* genomes, G + C content of the W25 was approximately 1.0% higher than other *C. ulcerans* strains (Busch et al. 2019). It may reflect significant variations in the gene content and virulence properties of this strain than other *C. ulcerans* isolates. Therefore, we compared the phenotypic and virulence properties of this strain and performed a comparative genomic analysis against other *C. ulcerans* isolates.

## Materials and methods

### Bacterial strains and culture conditions

Eight corynebacterial strains were included for phenotypic characterisation (Table 1). The strains were cultured in Brain Heart Infusion (BHI) broth at 37 °C and were incubated overnight in a shaking incubator.

**Table 1:** Bacterial strains used in this study

Strain	Description/source	References
*C. glutamicum* ATCC 13032	Soil (tox^−^)	Abe et al. (1967)
*C. diphtheriae* ISS3319	Human (tox^−^)	Sangal et al. (2015)
*C. diphtheriae* NCTC 10648	Human (tox^+^)	
*C. diphtheriae* NCTC 10356	Human nose (tox^−^)	
*C. ulcerans* 809	Human (tox^−^)	Dias et al. (2010)
*C. ulcerans* BR-AD22	Dog (tox^−^)	Mattos-Guaraldi et al. (2008)
*C. ulcerans* KL 756	Dog (tox^+^)	Möller et al. (2019)
*C. ulcerans* W25	Wild boar (tox^+^)	Busch et al. (2019)

### Strain identification

Strain W25 was analysed by MALDI-TOF mass spectrometry as previously described (Alibi et al. 2015). Biochemical tests were performed for strain W25 using the standard approach (Efstratiou and George 1999). Antimicrobial susceptibility testing method was carried out on Müller-Hinton agar as described in detail on the EUCAST website (http://www.eucast.org).

### Multiplex PCR

For differentiation of *Corynebacterium* species, a multiplex colony PCR, based on five genes, *rpoB*, *16S rRNA*, *pld*, *dtxR* and *tox*, was performed using the oligonucleotides listed in Table 2. For colony PCR, a loopful of freshly grown bacteria was resuspended in 500 μl of sterile deionized water and boiled for 10 min at 95 °C. The suspension was centrifuged at 13,000 × g for 1 min and 1 µl of the supernatant was used as template. Multiplex PCR was carried out in a Primus 96 advanced thermocyler (Peqlab, Erlangen) using previously described conditions (Torres Lde et al. 2013). The amplicons were separated by electrophoresis on a 3% agarose gel.

**Table 2:** Oligonucleotides used in this study

Name	Sequence (5′ → 3′)	Description
*RNA hybridization*		
DIP2222-s	GTCTCACTGAACCGTTGATG	Forward primer for *tox* gene
DIP2222-T7as	CCCGGGTAATACGACTCACTATAGGGCGCTATCGATAACTTGCGCAACG	Reverse primer for *tox* gene
16SrRNA-s	GCAGCCGCGGTAATACGTAG	Forward primer for *16S rRNA* gene
16SrRNA-as	GGGCCCTAATACGACTCACTATAGGGACATCT CACG ACAC GAGCTG	Reverse primer for *16S rRNA* gene
*Multiplex PCR*		
C2700 F	CGTATGAACATCGGCCAGGT	*rpoB*
C3130 R	TCCATTTCGCCGAAGCGCTG	*rpoB*
16S F	ACCGCACTTTAGTGTGTGTG	*16S rRNA*
16S R	TCTCTACGCCGATCTTGTAT	*16S rRNA*
pld F	ATAAGCGTAAGCAGGGAGCA	*pld*
pld R	TCAGCGGTGATTGTCTTCC	*pld*
dtxR 1F	GGGACTACAACGCAACAAGAA	*dtxR*
dtxR 1R	CAACGGTTTGGCTAACTGTA	*dtxR*
dipht 4F	GAACAGGCGAAAGCGTTAAGC	*tox*
dipht 4R	TGCCGTTTGATGAAATTCTTC	*tox*

### Detection of *C. diphtheriae* toxin production

Elek test (reaction of immunoprecipitation) was performed as described in the Manual for Diphtheria Laboratory Diagnosis (Efstratiou and George 1999; Mazurova et al. 1998). Korinetoksagar (State Research Center for Microbiology and Biotechnology, Obolensk, Russia) with 15% fetal calf serum was used to grow the bacterial strains. Filter paper strips (8.0 × 1.3 cm) were impregnated with 0.25 ml (500 IU in 1 ml) of purified diphtheria antitoxin (Microgen, Russia) and placed on the centre of the agar plates. Recommended control strains, toxigenic *C. diphtheriae* NCTC 10648, non-toxigenic *C. diphtheriae* NCTC 10356, and test cultures were transferred to the plate at a distance of 6-7 mm from the strip edge. The Elek test was analysed after 24 h of incubation at 37 °C.

### SDS-PAGE and Western blotting

*Corynebacterium ulcerans* strains were incubated at 37 °C in a shaking incubator in BHI broth (Oxoid, Wesel) and were grown to an OD_600_ of 0.4–0.6. For toxin production, 2–2′-bipyridyl was added at a final concentration of 0.5 mM during exponential phase and bacterial strains were incubated for further 2 h under iron starvation conditions (Moreira et al. 2003). The cells were harvested by centrifugation. Protein extraction, separation of proteins by SDS gel electrophoresis and immuno-detection of diphtheria toxin with human serum were carried out as described previously (Möller et al. 2019).

### RNA isolation and hybridization

RNA isolation and hybridization was carried out as described previously (Ott et al. 2010). For hybridization of the digoxigenin-labelled RNA probes (prepared by PCR using the oligonucleotides listed in Table 2) and detection, alkaline phosphatase-conjugated anti-digoxigenin Fab fragments and CSPD [disodium 3-(4-methoxyspiro{1,2-dioxetane-3,2′-(5′-chloro)tricyclo [3.3.1.13,7]decan}4-yl)phenyl phosphate] (Roche, Mannheim) were used. Chemiluminescence was detected using a ChemiDoc XRS + system (BioRad, Munich).

### Genome sequences

A scaffold was generated from the draft assembly of strain W25 (Accession number: VFEM00000000) against the genome of *C. ulcerans* strain PO100/5 using MeDuSa web-server (Bosi et al. 2015). The genome sequences of 28 other *C. ulcerans* strains and type strains of closely related corynebacterial species, *C. diphtheriae*, *Corynebacterium belfanti* and *Corynebacterium pseudotuberculosis* were obtained from the GenBank (Supplementary Table 1).

### Phylogenomic analyses

16S rRNA gene sequence (1509 bp in size) was extracted from the genome sequence of strain W25 using RNAmmer v1.2 (Lagesen et al. 2007). The reference 16S rRNA gene sequences of all corynebacterial strains were obtained from the GenBank. The nucleotide sequences were aligned using MUSCLE (Edgar 2004) and a phylogenetic tree was constructed from resulting sequence alignment (1155 bp in size after excluding sites with gaps) using IQtree with 100,000 ultra-fast bootstraps and 100,000 SH-aLRT tests (Nguyen et al. 2015). The tree was visualised using iTOL (Letunic and Bork 2016). Pairwise average nucleotide identities (ANI) were calculated among *C. ulcerans* genome sequences and the type strains of closely related corynebacterial species using FastANI (Jain et al. 2018). Digital DNA-DNA hybridisation (dDDH) values were calculated using Genome-to-Genome Distance Calculator 2.1 (Auch et al. 2010a, b). The genome sequence of strain W25 was also analysed using the PathoBacTyper (Tsai, Liu and Soo 2017), TrueBac™ ID cloud system (Ha et al. 2019) and Type (Strain) Genome Server (Meier-Kolthoff and Göker 2019).

All genome sequences were annotated using Prokka v 1.12 (Seemann 2014) and compared using Roary v 3.12.0 with an identity cut-off of 70% (Page et al. 2015; Tange 2011). A maximum-likelihood tree was calculated from the core genomic sequence alignment after removing the sites with missing data using IQ-Tree with 100,000 ultra-fast bootstraps and 100,000 SH-aLRT tests (Nguyen et al. 2015).

### Identification of virulence genes

The known virulence genes from pathogenic corynebacteria including *C. diphtheriae*, *C. pseudotuberculosis* as well as *C. ulcerans* were searched into the genome of strain W25 using the protein BLAST-searches (Altschul et al. 1997; Camacho et al. 2009).

### Identification of genes involved in starch metabolism

Glycoside hydrolases that are responsible for hydrolysis of amylose and amylopectin were identified from the KEGG pathway for starch and sucrose metabolism (https://www.genome.jp/kegg-bin/show_pathway?map00500) and were searched among the protein sequences of strains W25, PO100/5 and KL1196, *C. ulcerans* strains BR-AD22 and NCTC 12077, and *C. pseudotuberculosis* DSM 20689 obtained from the GenBank (Supplementary Table 1) using protein–protein PSI-BLAST algorithm (Altschul et al. 1997). A “KEGG-inferred” database was created with identified glycoside hydrolases sequences.

A two-step protein BLAST search strategy was applied to refine these results and to identify enzymes conserved among all corynebacterial strains. In the first search, proteins sequences from all six strains were searched in the “KEGG-inferred” database using BLASTP (Camacho et al. 2009). The query sequences with significant similarity to sequences in the database (≥ 90% coverage, ≥ 60% identity and e-value ≤ 1e-165) were aligned using Clustal-Omega (Sievers et al. 2011). The hierarchical clustering in the multiple sequence alignment allowed distinguishing protein groups, which were used to record a presence or absence of enzymes among individual strains. In the step 2, the proteins from each hierarchical cluster in the sequence alignment was used as the query against the entire proteome of the six corynebacterial strains. This was an additional confirmation of the presence or absence of a given enzyme in particular strains.

## Results

### Identification and biochemical characteristics of strain W25

**Fig. 1:**
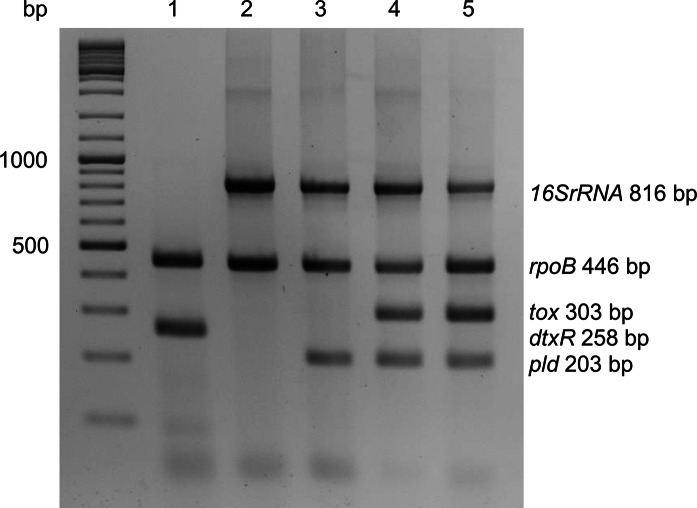
Multiplex PCR for the identification of corynebacteria. Lane 1: *C. diphtheriae* ISS 3319 (tox^−^); 2: *C. ulcerans* 809 (tox^−^); 3: *C. ulcerans* BR-AD22 (tox^−^); 4: *C. ulcerans* KL756 (tox^+^); 5: *C. ulcerans* W25

Strain W25 was initially identified by MALDI-TOF mass spectrometry as *C. ulcerans* with a score of 2.065. Multiplex PCR amplified fragments of 16S rRNA, *rpoB* and *tox* genes with a faint DNA band for *pld* gene for strain W25 (Fig. 1), a profile consistent with other *C. ulcerans* isolates as the primers were designed to amplify the fragments of *rpoB* and *tox* genes for *C. diphtheriae*, *C. pseudotuberculosis* and *C. ulcerans*, 16S rRNA for *C. ulcerans* and *C. pseudotuberculosis*, *pld* for *C. pseudotuberculosis* and *dtxR* for *C. diphtheriae* strains.

Isolate W25 was found to produce H_2_S on Tinsdale medium when stabbed into the surface and was urease-positive (Table 3). The strain was positive for reverse CAMP reaction, i.e., produced phospholipase D inhibiting β-haemolysis by *Staphylococcus aureus* on blood-agar plates. The strain was also positive for hydrolase activity and was able to utilise glucose as a carbon source (Table 3). W25 could not hydrolyse gelatine, reduce nitrate or ferment starch and was negative for toxin production according to the Elek test (Table 3). The strain was sensitive to all antibiotics tested (Supplementary Table 2). The extensive search for glycoside hydrolases revealed the absence of two enzymes 1,4-alpha-amylase and a type I pullulanase, in the strain W25. These enzymes are involved in starch hydrolysis and an absence of these enzymes explains inability of W25 strain to ferment starch (Table 4).

**Table 3:** Biochemical characteristics of strain W25

Strains	Elek test	H_2_S	Nitrate reductase	Urease	Reverse camp	DNAse activity	Gelantine hydrolysis	Starch	Glucose
C. diphtheriae^*^	±	+	±	–	–	+	–	±	+
C. ulcerans^*^	±	+	–	+	+	+	±	+	+
C. pseudotuberculosis^*^	±	+	±	+	+	–	±	±	+
Strain W25	–	+	–	+	+	+	–	–	+

*Expected results taken from Bernard and Funke (2015), Dorella et al. (2006), and Mattos-Guaraldi et al. (2014)

**Table 4:** A presence and absence of two enzymes involved in starch metabolism

Protein name	Strain	NCBI Protein ID
Pullulanase type I (Protein cluster: PCLA_2760914)	*C. ulcerans* BR-AD22	AEG83474.1
	*C. ulcerans* NCTC12077	ESU58386.1
	W25	Absent
	PO100/5	Absent
	KL1196	Absent
	*C. pseudotuberculosis* DSM20689	RKT29492.1
1,4-Alpha amylase (Protein cluster: PCLA_3428021)	*C. ulcerans* BR-AD22	AEG82814.1
	*C. ulcerans* NCTC12077	ESU59224.1
	W25	Absent
	PO100/5	Absent
	KL1196	Absent
	*C. pseudotuberculosis* DSM20689	Absent

### Phylogenomic characterisation

**Fig. 2:**
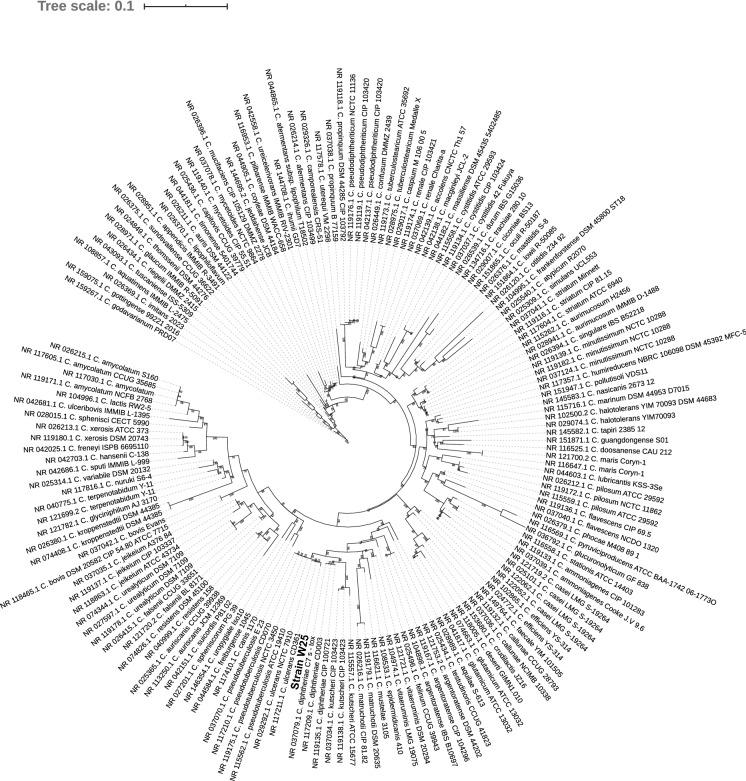
Maximum likelihood tree from the alignment of 16S rRNA sequences for all *Corynebacterium* species. The scale bar represents nucleotide substitution per site

The maximum-likelihood tree from 16S rRNA gene also grouped strain W25 *with C. ulcerans* isolates (Fig. 2). 16S rRNA sequence of strain W25 showed 99.65% similarity with the 16S rRNA gene in *C. ulcerans* strain CD361 and 99.31% similarity with the 16S rRNA gene of *C. ulcerans* strain NCTC 7910.

**Fig. 3:**
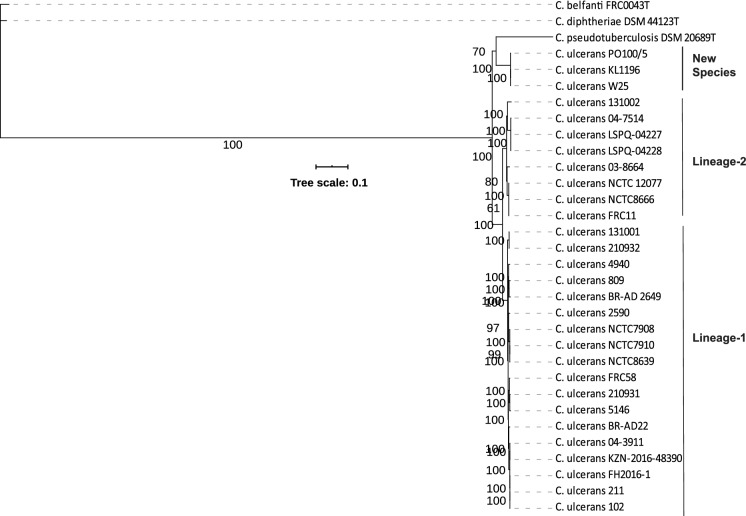
Maximum likelihood tree from the core genome alignment. The scale bar represents nucleotide substitution per site

The results of core genome phylogeny showed that W25 has separated from other *C. ulcerans* genomes and formed a distinct cluster with two other strains, PO100/5 and KL1196 (Fig. 3). The latter two strains are also submitted to the GenBank as *C. ulcerans* isolates. The remaining 26 *C. ulcerans* strains formed two distinct subgroups, which is in agreement with our previous study showing an existence of two lineages within this species (Subedi et al. 2018). The composition of these lineages is highly consistent between the two studies with five additional strains (211, FH2016-1, NCTC 7908, NCTC 7910 and NCTC 8639) grouped in lineage 1 and two additional strains (03-8664 and NCTC 8666) grouped in lineage 2 (Fig. 3) in this study.

Pairwise dDDH values of strain W25 with other *C. ulcerans* isolates and type strains of *C. belfanti*, *C. diphtheriae* and *C. pseudotuberculosis*, indicate that strain W25 belong to a novel species along with PO100/5 and KL1196 (Table 5). These results are also confirmed by the ANI values. The ANI values between strains W25, PO100/5 and KL1196 were > 99%, consistent with them being the same species but were < 92% between these strains and *C. ulcerans* genomes (Supplementary Table 3).

**Table 5:** dDDH values using recommended formula #2 between strain W25 and other *C. ulcerans* strains and type strains of *C. belfanti*, *C. diphtheriae* and *C. pseudotuberculosis*

Reference	DDH	Model C.I.	Distance	G + C difference
*C. diphtheriae* DSM 44123^T^	22.1	[19.8–24.5%]	0.1986	0.91
*C. belfanti* FRC0043^**T**^	22.6	[20.4–25.1%]	0.1935	0.81
*C. pseudotuberculosis* DSM 20689^**T**^	28.5	[26.2–31%]	0.1505	2.25
NCTC 7910^**T**^	40.9	[38.4–43.5%]	0.0964	1.12
809	41	[38.5–43.5%]	0.0961	1.13
BR-AD22	40.9	[38.4–43.5%]	0.0964	1.04
102	40.9	[38.4–43.5%]	0.0964	1.08
NCTC 12077	41	[38.5–43.6%]	0.096	1.05
FRC58	40.9	[38.4–43.5%]	0.0964	1.13
210932	41	[38.5–43.5%]	0.0962	1.12
210931	40.7	[38.3–43.3%]	0.097	1.13
FRC11	41.3	[38.8–43.9%]	0.0951	1.09
5146	40.9	[38.4–43.5%]	0.0964	1.13
131002	41.6	[39.1–44.1%]	0.0943	1.06
LSPQ-04227	41.5	[39–44%]	0.0946	1.04
LSPQ-04228	41.5	[39–44%]	0.0946	1.04
131001	41	[38.5–43.5%]	0.0962	1.12
04-3911	41	[38.5–43.5%]	0.0963	1.11
03-8664	42.1	[39.6–44.6%]	0.0927	0.96
04-7514	41.2	[38.7–43.7%]	0.0956	0.97
KZN-2016-48390	40.9	[38.4–43.5%]	0.0964	1.05
BR-AD 2649	41.1	[38.6–43.6%]	0.0958	1.17
2590	40.8	[38.3–43.3%]	0.0969	1.14
4940	41	[38.5–43.5%]	0.0963	1.09
211	40.9	[38.4–43.5%]	0.0964	1.08
FH2016-1	40.9	[38.5–43.5%]	0.0963	1.08
NCTC 8666	41.2	[38.7–43.7%]	0.0956	1.05
NCTC 7908	40.9	[38.4–43.5%]	0.0964	1.12
NCTC 8639	40.9	[38.4–43.5%]	0.0964	1.12
PO100/5	98.5	[97.7–99%]	0.0025	0.04
KL1196	100	[100–100%]	0	0

An analysis of the genome sequence of strain W25 using PathoBacTyper (Tsai et al. 2017) showed 29% coverage rate against *Corynebacterium variabile* strain and Type (Strain) Genome Server (Meier-Kolthoff and Göker 2019) indicated that this strain belongs to a novel species. Similarly, TrueBac™ ID cloud system revealed *C. ulcerans* to be the closest species with 90.85% ANI (90.6% ANI coverage), 99.72% similarity between the 16S rRNA, 94.95% similarity between *recA* and 99.09% sequence similarity between *rplC* genes of the two strains. These results indicate that strains W25 together with PO100/5 and KL1196 should be separated from *C. ulcerans* as a novel species.

A comparative genomic analysis of all *C. ulcerans* and W25, PO100/5 and KL1196 strains using Roary (Page et al. 2015) with a minimum BLASTP identity of 70%, revealed a pangenome encompassing 4525 genes, of which 1,555 genes belonged to the core genome. The number of genes on individual genomes varied between 2159 and 2529; therefore, > 61% of the genome is conserved between the two species. Only 30 genes were found to be unique to *C. ulcerans* strains including 23 genes encoding hypothetical proteins (Supplementary Table 4). Homologs of six of the remaining seven genes encoding aminopeptidase N, cysteine-tRNA ligase, 1,4-dihydroxy-2-naphthoyl-CoA synthase, heat-inducible transcription repressor HrcA, putative fluoride ion transporter CrcB and putative propionyl-CoA carboxylase beta chain 5 (AccD5) are also present among strains of the novel group. However, RNA polymerase sigma factor YlaC appears to be unique to *C. ulcerans* strains. In contrast, 238 genes were unique to strains W25, PO100/5 and KL1196 (Supplementary Table 5) that were absent among *C. ulcerans* isolates. Again, 92% (220 genes) of these genes encode hypothetical/putative proteins. Some of the unique genes encoding ABC transporter ATP-binding proteins, a UDP-glucose 6-dehydrogenase, glucose-specific EIIA component of phosphotransferase system, proteins involved in hemin transport system (HmuU), vitamin B12 import system (BtuC) and resistance to daunorubicin/doxorubicin (DrrA) have other copies or homologs that are conserved across all the strains and may not cause any functional variation between *C. ulcerans* strains and those belong to the novel group. Similarly, copies of genes encoding sulfate/thiosulfate import ATP-binding protein (CysA) is present among some *C. ulcerans* strains. Eight genes encoding component of ammonia channel (*amt*), a *bgl* operon antiterminator (BglG), D-amino acid dehydrogenase (DadA), glucose-specific EIICBA components of phosphotransferase system, oligopeptide transport system permease protein (OppB), a putative peptidase (cp29_00169) and a transcriptional regulatory protein DesR are unique to strains W25, PO100/5 and KL1196 and may be responsible for minor functional variations between these species (Supplementary Table 5).

### Expression of the *tox* gene in strain W25

Diphtheria toxin is the main virulence factor in toxigenic corynebacteria (Sangal and Hoskisson 2014). The *tox* gene has been amplified in the multiplex PCR reaction (Fig. 1).

**Fig. 4:**
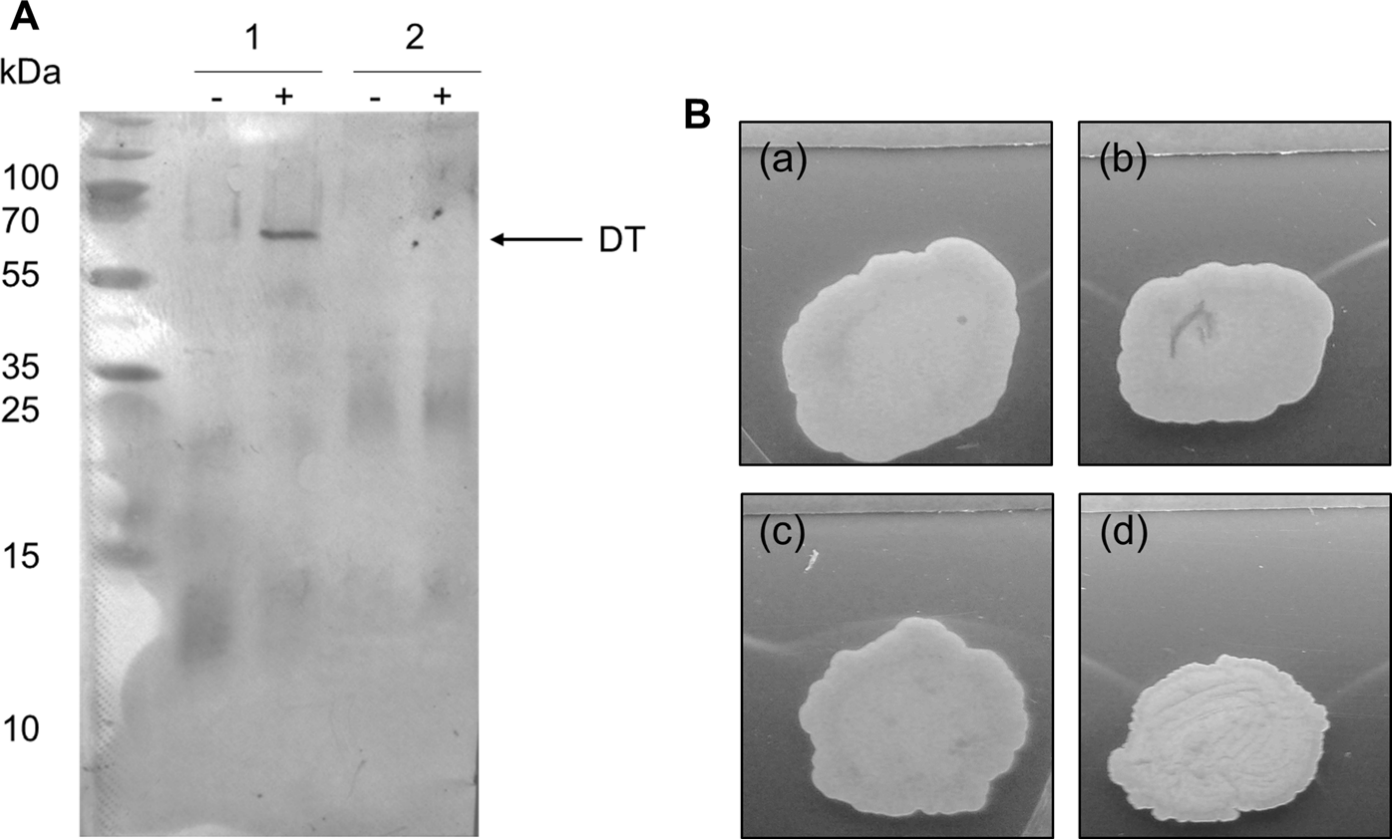
Detection of diphtheria toxin. **A** Western blot analysis using DT-specific antiserum and cell extracts from toxigenic *C. ulcerans* KL756 and from strain W25. Bacteria were grown without (−) bipyridyl for control and with (+) this iron chelator to induce iron starvation and induction of *tox* transcription. 2 µg of protein extract were added per lane. Human serum collected 1 year after primary and two booster vaccinations (second serum) was used as primary antibody. 1: KL756 (tox^+^), 2: W25. The arrow indicates DT with an apparent molecular mass of 62 kDa. **B** Elek test with purified antitoxin. (a): *C. ulcerans* 809 (tox^−^); (b): *C. ulcerans* BR-AD22 (tox^−^); (c): *C. ulcerans* KL756 (tox^+^); (d): *C. ulcerans* W25. As a control *C. diphtheriae* strains NCTC 10648 (positive control) and NCTC 10356 (negative control) were used. After an incubation of 24 h, precipitation lines for the toxigenic *C. ulcerans* strain KL756 were detected

A Western blot using human antiserum against the toxin was negative for strain W25 (Fig. 4A). Diphtheria toxin was detected in extract from strain KL756 during the induction of iron starvation (Fig. 4A). We also performed an Elek test to check the expression of the gene. Elek test is an agar gel immunodiffusion assay where horse diphtheria antitoxin diffuses towards diphtheria toxin produced by toxigenic strains. A precipitation line forms near the bacterial colonies at the zone of equivalence. No precipitation lines were observed for W25, suggesting that this strain is non-toxigenic (Fig. 4B).

A BLAST-search of the protein sequence of the toxin from *C. diphtheriae* NCTC 13129 (DIP0222) showed significant similarity with the protein encoded by the gene cp29_02234 in strain W25 that was annotated to encode a hypothetical protein. A nucleotide sequence alignment of the *tox* gene including 100 bp upstream and 100 bp downstream regions from strains W25, PO100/5, KL1196, *C. diphtheriae* NCTC 13129 (*DIP0222*) and *C. ulcerans* 0102 (*CULC0102_0213*) revealed that the gene in strains W25 and KL1196 has a two base (GG) insertion at position 48 (Supplementary Fig. 1), which introduced a frameshift, leading it to be a pseudogene. Therefore, W25 strain is an NTTB strain, which is consistent with nucleotide sequence being amplified in a PCR reaction and Elek test being negative.

**Fig. 5:**
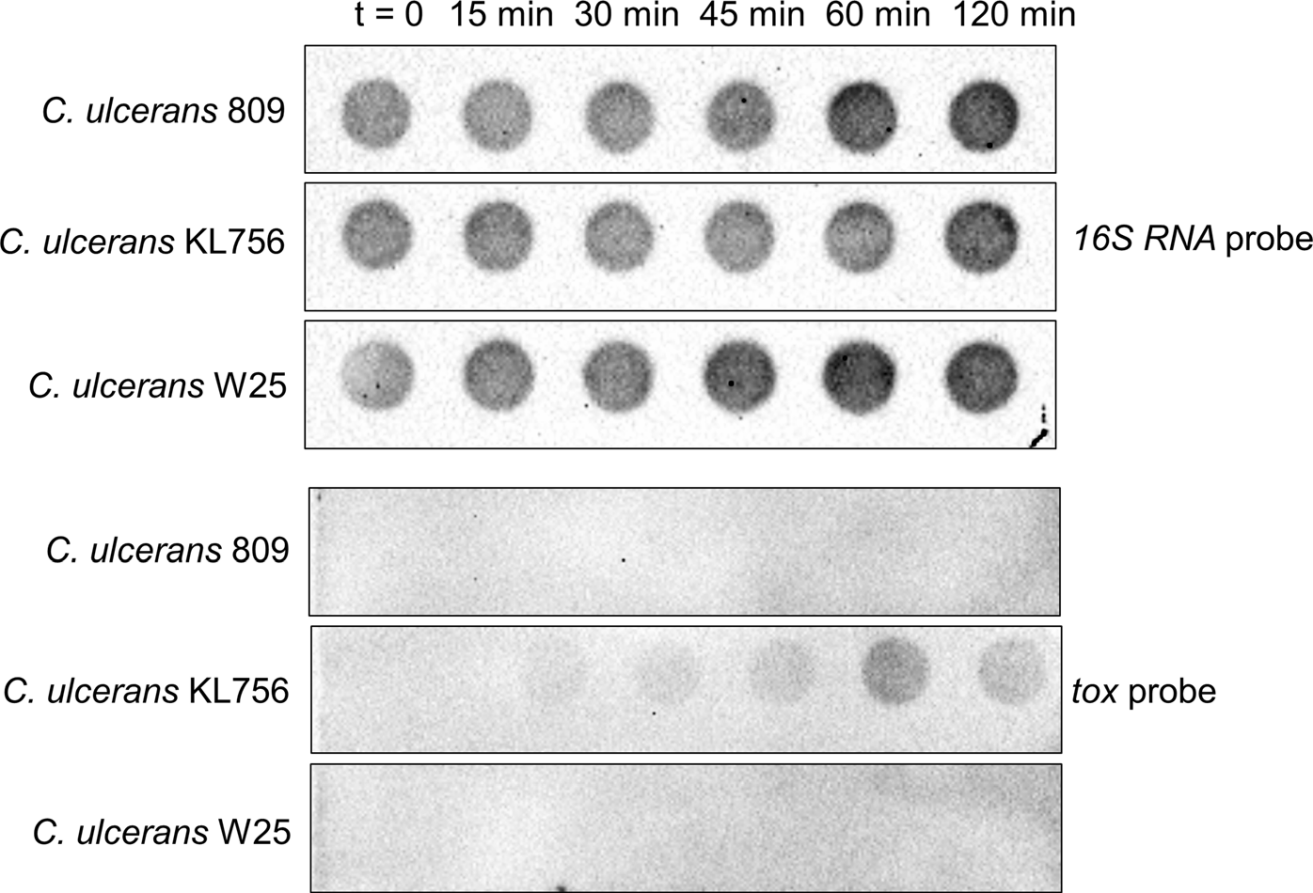
Transcription of the *tox* gene. RNA hybridization of *C. ulcerans* strains (tox^−^: 809; tox^+^: KL756) and W25 at six different time points (t = 0: before induction with bipyridyl; 15, 30, 45, 60 and 120 min post induction). A *16SrRNA* probe was used as a control. RNA hybridization experiments were carried out in three independent biological replicates

To further test the toxin expression, RNA hybridization experiments were carried out using tox^+^*C. ulcerans* strain KL756 as the positive control and tox^−^ strain 809 as the negative control. A presence of *16S rRNA* gene expression was confirmed in all three strains (Fig. 5A) whereas transcript for *tox* gene was only detected for strain KL756 when iron starvation was induced by bipyridyl (Fig. 5B). These results confirmed that W25 is an NTTB strain.

### Pilus genes clusters in strain W25

**Fig. 6:**
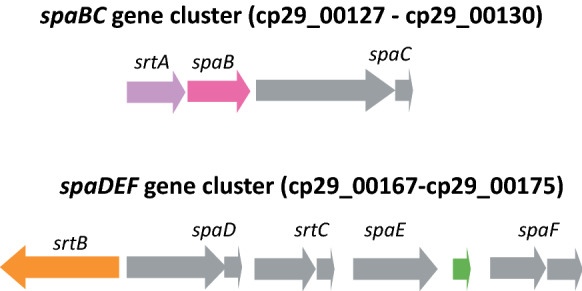
*spa* gene clusters in strain W25. The schematic is not to scale. The direction of arrows indicate the orientation of the coding sequence

Similar to *C. ulcerans* strains, two *spaBC* and *spaDEF* type pilus gene clusters have been identified in strain W25 (Subedi et al. 2018; Trost et al. 2011). The genes encoding sortase A and SpaB fimbrial subunit are present in strain W25; however, the C-terminal region of the SpaC fimbrial subunit is truncated and the corresponding gene is annotated as two smaller genes (Fig. 6). Therefore, SpaBC type pili in this strain may not be functional.

Similarly, *spaD*, *srtC* and *spaF* genes are also truncated and annotated as two smaller genes, respectively, and SpaE is missing approximately 65 amino acid residues at the N-terminal region. A small gene encoding hypothetical protein is also present between *spaE* and *spaF* (Fig. 6). Therefore, SpaDEF type pili may also be absent in strain W25.

### Other virulence genes in strain W25

When searched for other corynebacterial virulence genes, strain W25 was found to possess most of the other virulence genes present in *C. ulcerans* strains including *cpp* (corynebacterial protease), *pld* (phospholipase D), *nanH* (neuraminidase, sialidase), *vsp1* and *vsp2* (trypsin-like serine protease) and *cwlH* (hydrolase; cell wall peptidase; Table 6). A gene annotated as *ripA* (peptidoglycan endopeptidase) show 91% identities with the *rpfI* gene in *C. ulcerans* with a deletion of 34 amino acids from position 299 to 332. However, two virulence genes *rbp* and *tspA* were absent in strain W25.

**Table 6:** Other virulence genes in strain W25

Gene	Gene in W25	Protein
*pld*	cp29_02424	Phospholipase D
*nanH*	cp29_01937	Neuraminidase (sialidase)
*vsp1*	cp29_01856	Trypsin-like serine protease
*ripA*	cp29_01144	Peptidoglycan endopeptidase
*cwlH*	cp29_00749	Hydrolase (cell wall peptidase)
*vsp2*	cp29_00148	Protease
*cpp*	cp29_00135	Protease (endo-beta-N-acetylglucosaminidase F2)
*cpfrc_00397*	cp29_01926	Type VII secretion-associated serine protease mycosin
*dtsR2*	cp29_01827	AccD5-3 acyl-CoA carboxylase subunit beta
*dtsR1*	cp29_01826	AccD5-2 propionyl-CoA carboxylase beta chain 2
*accD3*	cp29_00074	AccD5-1 acyl-CoA carboxylase subunit beta
*cpfrc_00536*	cp29_01777	Hydrolase
*nrpS1*	cp29_01752	Non-ribosomal peptide synthetase
*rfpA*	cp29_01715	Resuscitation-promoting factor
*rfpB*	cp29_01628	Resuscitation-promoting factor

Strain W25 also possessed several virulence genes identified in *C. pseudotuberculosis* strain FRC41 (Trost et al. 2010). These include *cpfrc_00397* (secretion-associated serine protease), *dtsR1* (acyl-CoA carboxylase subunit beta), *dtsR2* (acyl-CoA carboxylase subunit beta), *accD3* (propionyl-CoA carboxylase beta chain 2), *cpfrc_00536* (secreted SGNH-hydrolase), *nrpS1* (nonribosomal peptide synthetase 1) as well as *rpfA* and *rpfB* (resuscitation-promoting factors). However, two genes *nor* (nitric oxide reductase) and *cpfrc_00562* (secreted trypsin-like serine protease) were found to be absent.

## Discussion

*C. ulcerans* and *C. pseudotuberculosis* are pathogens adapted to canine and ovine hosts, respectively but can cause zoonotic infections in humans (Bregenzer et al. 1997; Hacker et al. 2016; Peel et al. 1997). In this study, we have characterised an isolate W25, which has been identified as an atypical *C. ulcerans* based in MALDI-TOF analysis, multiplex PCR using 16S rRNA, *rpoB* and *tox* genes (Fig. 1) and other biochemical characteristics (Table 3).

Genome-based matrices have been extensively used to define novel bacterial species (Nouioui et al. 2018, 2019; Sangal et al. 2016, 2018). The dDDH and ANI cut-off values for defining new species are 70% and 95%, respectively (Auch et al. 2010b; Konstantinidis and Tiedje 2005, 2007). The genome sequences of strains W25 showed > 98% dDDH and > 99% ANI values against the genome sequences of strains PO100/5 and KL1196, clearly indicating that these strains belong to the same species. These strains are phylogenetically closely related to other pathogenic corynebacteria, *C. diphtheriae*, *C. pseudotuberculosis* and *C. ulcerans* (Figs. 2 and 3). However, dDDH and ANI values were below the species cut-off between W25 and the type strains of these species (Table 5; Supplementary Table 3), suggesting that this strain belong to a novel *Corynebacterium* species along with two other strains PO100/5 and KL1196. These results are also confirmed by other bacterial identification platforms including PathoBacTyper (Tsai et al. 2017), TrueBac™ ID cloud system (www.truebacid.com) and Type (Strain) Genome Server (Meier-Kolthoff and Göker 2019). W25 was isolated from a case of necrotizing lymphadenitis in a wild boar (Busch et al. 2019), PO100/5 from a pig and KL1196 from a roe deer. Therefore, this species also appears to be prevalent among animals.

One of the key biochemical difference separating strain W25 from *C. ulcerans* was inability of the former to ferment starch (Table 3). We identified protein sequences for pullulanase type I and 1,4-alpha amylase enzymes that are involved in starch metabolism are absent in strain W25 as well as PO100/5 and KL1196 (Table 4). The genes encoding these enzymes are inactive (pseudogenes) due to frameshift mutations among these isolates. While both the genes are present in *C. ulcerans*, the gene encoding 4-alpha amylase enzyme is absent in *C. pseudotuberculosis* (Table 4). In general, *C. pseudotuberculosis* strains do not ferment starch (Dorella et al. 2006). Therefore, both the enzymes seem to be important for starch metabolism and an absence of any of these may compromise the ability to ferment starch.

Interestingly, the genome of strain W25 has been annotated to carry the *tox* gene, encoding a diphtheria-like toxin but the protein was not detectable in the Western blot using human antiserum against the toxin (Fig. 4A) or in the Elek test (Fig. 4B). Furthermore, no transcript of the *tox* gene was observed in the RNA hybridization experiment (Fig. 5A, B). The gene has a two base (GG) insertion at position 48 which has introduced the frameshift (Supplementary Fig. 1).

Two pilus gene clusters (*spaBC* and *spaDEF*) have been identified in strain W25; however, both of them show potential loss of the gene functions (Fig. 6). A *spaBC* cluster is also present in *C. ulcerans*, which lacks the *spaA* gene encoding a major pilin subunit (Subedi et al. 2018; Trost et al. 2011). SpaABC type pili are known to interact with pharyngeal epithelial cells (Mandlik et al. 2007; Reardon-Robinson and Ton-That 2014) and homodimeric or heterodimeric SpaB/SpaC proteins were suggested to facilitate this interaction (Trost et al. 2011). However, *spaC* gene encoding the tip protein is also truncated in strain W25. Similarly, multiple genes of the the *spaDEF* cluster are truncated in strain W25 (Fig. 6). These pili in *C. diphtheriae* are characterised to interact with laryngeal epithelial cells (Mandlik et al. 2007; Reardon-Robinson and Ton-That 2014). The *spaDEF* cluster is characterised of five genes, *spaD*, *spaE* and *spaF* encoding the major pilin subunit, minor subunit and the tip protein, respectively and two sortases-encoding genes, *srtB* and *srtC*, responsible assembly of the pilus (Mandlik et al. 2007; Reardon-Robinson and Ton-That 2014). Therefore, it is possible that the ability of this strain to interact with the pharyngeal or laryngeal epithelial cells is compromised.

However, the strain W25 possesses a number of virulence genes present among *C. ulcerans* and *C. pseudotuberculosis* strains, which may enable it to cause severe invasive infections (Table 6). For example, phospholipase D is a well-characterised virulence-associated protein responsible for significant macrophage death (McKean et al. 2007). Similarly, protease and hydrolases activities of other proteins have been found to contribute to the virulence properties in pathogenic corynebacteria (Trost et al. 2010, 2011).

## Conclusions

Isolate W25 is biochemically similar to *C. ulcerans* strains that can produce H_2_S on Tinsdale medium, is positive for reverse CAMP reaction and hydrolase activity, and is able to utilise glucose as a carbon source (Table 3). This strain was previously defined as atypical *C. ulcerans* but belong to a novel species including two strains, PO100/5 and KL1196, which were independently isolated from animals. The isolate is likely an NTTB strain with compromised abilities to adhere to pharyngeal and laryngeal epithelial cells due to loss of the multiple genes in *spaBC* and *spaDEF* pilus gene clusters. However, a number of corynebacterial virulence genes are present, which may enable the strain to cause severe invasive infections in animals and zoonotic infections in humans.

## Electronic supplementary material

Below is the link to the electronic supplementary material.

Supplementary material 1 (DOCX 15 kb)

Supplementary material 2 (DOCX 12 kb)

Supplementary material 3 (PDF 87 kb)

Supplementary material 4 (PDF 207 kb)

Supplementary material 5 (DOCX 13 kb)

Supplementary material 6 (DOCX 22 kb)
