# Supplementary material for: Phylogenomic characterisation of a novel corynebacterial species pathogenic to animals
Source: Antonie Van Leeuwenhoek. 2020 Jun 4;113(8):1225–39. doi: 10.1007/s10482-020-01430-5 (PMC7334274; doi:10.1007/s10482-020-01430-5)
Supplement: Supplementary file 1 — Supplementary material 1 (DOCX 15 kb) [file 10482_2020_1430_MOESM1_ESM.docx]

Supplementary Table 1. List of genome obtained from the GenBank for comparative analyses

| **Organism** | **Strain** | **Assembly** | **Size (Mb)** | **GC%** | **Accession No** | **#contigs** |
| --- | --- | --- | --- | --- | --- | --- |
| *Corynebacterium ulcerans* | 809 | GCA_000215645.1 | 2.5021 | 53.3 | NC_017317.1 | 1 |
| *Corynebacterium ulcerans* | BR-AD22 | GCA_000215665.1 | 2.60637 | 53.4 | NC_015683.1 | 1 |
| *Corynebacterium ulcerans* | 102 | GCA_000306825.1 | 2.57919 | 53.4 | NC_018101.1 | 1 |
| *Corynebacterium ulcerans* | NCTC 12077 | GCA_000498915.1 | 2.61629 | 53.4 | AYUJ01 | 15 |
| *Corynebacterium ulcerans* | FRC58 | GCA_000499805.2 | 2.5426 | 53.3 | NZ_CP011913.1 | 1 |
| *Corynebacterium ulcerans* | 210932 | GCA_000767415.1 | 2.48434 | 53.3 | NZ_CP009500.1 | 1 |
| *Corynebacterium ulcerans* | 210931 | GCA_000767645.1 | 2.50943 | 53.2536 | NZ_CP009583.1 | 2 |
| *Corynebacterium ulcerans* | FRC11 | GCA_000767685.1 | 2.44283 | 53.3 | NZ_CP009622.1 | 1 |
| *Corynebacterium ulcerans* | 5146 | GCA_000769635.1 | 2.46644 | 53.3 | NZ_CP009716.1 | 1 |
| *Corynebacterium ulcerans* | 131002 | GCA_000968945.1 | 2.43457 | 53.4 | NZ_CP011095.1 | 1 |
| *Corynebacterium ulcerans* | LSPQ-04227 | GCA_000988285.1 | 2.42822 | 53.4 | JZUS01 | 10 |
| *Corynebacterium ulcerans* | LSPQ-04228 | GCA_000988295.1 | 2.43938 | 53.4 | JZUT01 | 23 |
| *Corynebacterium ulcerans* | 131001 | GCA_001281445.1 | 2.48332 | 53.3 | NZ_CP010818.1 | 1 |
| *Corynebacterium ulcerans* | 04-3911 | GCA_001298275.1 | 2.49268 | 53.3 | LGSX01 | 40 |
| *Corynebacterium ulcerans* | 03-8664 | GCA_001298285.2 | 2.42868 | 53.5 | LGSY02 | 258 |
| *Corynebacterium ulcerans* | 04-7514 | GCA_001302345.1 | 2.49784 | 53.5 | LJVH01 | 28 |
| *Corynebacterium ulcerans* | KZN-2016-48390 | GCA_001876325.1 | 2.54111 | 53.4 | MIOS01 | 31 |
| *Corynebacterium ulcerans* | PO100/5 | GCA_002162115.1 | 2.57241 | 54.4 | NZ_CP021417.1 | 1 |
| *Corynebacterium ulcerans* | BR-AD 2649 | GCA_002866485.1 | 2.54148 | 53.3 | MPST01 | 17 |
| *Corynebacterium ulcerans* | 2590 | GCA_002866525.1 | 2.50137 | 53.3 | MPSS01 | 10 |
| *Corynebacterium ulcerans* | 4940 | GCA_002872395.1 | 2.41937 | 53.3 | LSWN01 | 21 |
| *Corynebacterium ulcerans* | KL1196 | GCA_004382105.1 | 2.55035 | 54.4 | SDVD01 | 20 |
| *Corynebacterium ulcerans* | 211 | GCA_008995395.1 | 2.57908 | 53.4 | NZ_AP019662.1 | 1 |
| *Corynebacterium ulcerans* | FH2016-1 | GCA_009002285.1 | 2.57913 | 53.4 | NZ_AP019663.1 | 1 |
| *Corynebacterium ulcerans* | NCTC7910T | GCA_900187135.1 | 2.45376 | 53.3 | NZ_LT906443.1 | 1 |
| *Corynebacterium ulcerans* | NCTC8666 | GCA_900447245.1 | 2.54241 | 53.4 | UFXR01 | 2 |
| *Corynebacterium ulcerans* | NCTC7908 | GCA_900475635.1 | 2.45367 | 53.3 | NZ_LS483400.1 | 1 |
|  |  |  |  |  |  |  |
| ***Organism*** | **Strain** | **Assembly** | **Size (Mb)** | **GC%** | **Accession No** | **#contigs** |
| *Corynebacterium ulcerans* | NCTC8639 | GCA_900475775.1 | 2.45375 | 53.3 | NZ_LS483416.1 | 1 |
| *Corynebacterium ulcerans* | W25 | GCA_006370535.1 | 2.55092 | 54.4 | VFEM01 (new) | 1 |
| *Corynebacterium belfanti* | FRC0043T | GCA_900205605.1 | 2.60942 | 53.6 | OANN01.1 | 156 |
| *Corynebacterium diphtheriae* | DSM 44123T | GCA_001913265.1 | 2.3716 | 53.5 | LJXR01.1 | 28 |
| *Corynebacterium pseudotuberculosis* | DSM 20689T | GCA_003634885.1 | 2.33855 | 52.2 | RBXH01.1 | 1 |
