# Supplementary material for: Phylogenomic characterisation of a novel corynebacterial species pathogenic to animals
Source: Antonie Van Leeuwenhoek. 2020 Jun 4;113(8):1225–39. doi: 10.1007/s10482-020-01430-5 (PMC7334274; doi:10.1007/s10482-020-01430-5)
Supplement: Supplementary file 2 — Supplementary material 2 (DOCX 12 kb) [file 10482_2020_1430_MOESM2_ESM.docx]

Supplementary Table 2. Antimicrobial resistance profile strain W25

| **Antibiotics** | **Concentration** | **Zone diameter (mm)** | **Susceptibility** | **Breakpoint (mm)** |
| --- | --- | --- | --- | --- |
| Penicillin G | 1 E | 32 | sensitive | 29 |
| Ampicillin | 2 µg | 35 | sensitive* | --- |
| Cefuroxim | 30 µg | 47 | sensitive* | --- |
| Ceftriaxon | 30 µg | 42 | sensitive* | --- |
| Meropenem | 10 µg | 50 | sensitive* | --- |
| Moxifloxacin | 5 µg | 43 | sensitive | 25 |
| Erythromycin | 15 µg | 46 | sensitive* | --- |
| Clindamycin | 2 µg | 34 | sensitive | 20 |
| Vancomycin | 5 µg | 22 | sensitive | 17 |
| Rifampicin | 5 µg | 50 | sensitive | 30 |

* No evaluation criteria (EUCAST)
