# Supplementary material for: Phylogenomic characterisation of a novel corynebacterial species pathogenic to animals
Source: Antonie Van Leeuwenhoek. 2020 Jun 4;113(8):1225–39. doi: 10.1007/s10482-020-01430-5 (PMC7334274; doi:10.1007/s10482-020-01430-5)
Supplement: Supplementary file 3 — Supplementary material 3 (PDF 87 kb) [file 10482_2020_1430_MOESM3_ESM.pdf]

Supplementary Table 3. Pairwise ANI values within the dataset.

Strain	809	BR-AD22	102	NCTC 12077	FRC58	210932	210931	FRC11	5146	131002	LSPQ-04227	LSPQ-0422	131001	04-3911	03-8664	04-7514	KZN-2016-4839	BR-AD 2649	2590	4940	211	FH2016-1	NCTC7910	NCTC8666	NCTC7908	NCTC8639	PO100/5	KL1196	W25	*C. belfanti* FRC0	*C. diphtheri*	*C. pseudotuberculosis* DSM 206
809	100.00	98.68	98.56	96.37	98.65	98.50	98.73	96.36	98.67	95.77	95.83	95.83	98.49	98.61	96.31	95.84	98.58	98.74	98.63	98.89	98.57	98.57	98.73	96.38	98.70	98.71	91.12	91.07	91.12	79.18	79.17	85.21
BR-AD22	98.74	100.00	98.88	96.25	98.58	98.58	98.65	96.34	98.70	95.75	95.82	95.80	98.58	98.93	96.29	95.80	98.91	98.50	98.65	98.71	98.88	98.89	98.67	96.26	98.70	98.70	91.10	91.10	91.05	79.35	79.31	85.08
102	98.55	98.87	100.00	96.34	98.91	98.73	98.60	96.35	98.93	95.75	95.75	95.74	98.72	99.93	96.34	96.01	99.84	98.31	98.54	98.61	100.00	99.99	98.66	96.23	98.63	98.67	91.11	91.13	91.05	79.41	79.24	84.97
NCTC 12077	96.38	96.25	96.31	100.00	96.34	96.37	96.31	99.97	96.38	97.82	98.03	98.04	96.40	96.28	98.46	98.00	96.34	96.16	96.33	96.38	96.33	96.33	96.36	99.81	96.35	96.39	91.13	91.26	91.14	79.43	79.39	84.99
FRC58	98.62	98.62	98.99	96.37	100.00	98.57	99.31	96.37	99.38	95.76	95.79	95.79	98.59	99.08	96.32	95.84	98.98	98.33	98.50	98.56	98.98	98.99	98.71	96.23	98.72	98.75	91.14	91.04	91.06	79.35	79.16	85.05
210932	98.45	98.56	98.73	96.36	98.61	100.00	98.49	96.35	98.59	95.86	95.88	95.85	99.99	98.72	96.35	95.90	98.69	98.31	98.53	98.60	98.71	98.72	98.61	96.33	98.65	98.64	91.15	91.07	91.09	79.22	78.95	85.03
210931	98.74	98.65	98.63	96.34	99.33	98.54	100.00	96.34	99.51	95.83	95.81	95.82	98.50	98.69	96.32	95.80	98.66	98.41	98.54	98.65	98.60	98.63	98.74	96.36	98.76	98.73	91.05	90.97	91.00	79.40	79.06	85.08
FRC11	96.38	96.29	96.31	99.96	96.34	96.37	96.34	100.00	96.40	97.85	98.05	98.05	96.36	96.38	98.52	98.06	96.35	96.23	96.31	96.36	96.30	96.32	96.42	99.97	96.42	96.43	91.21	91.21	91.26	79.26	79.31	85.10
5146	98.63	98.69	98.93	96.36	99.37	98.59	99.51	96.45	100.00	95.86	95.83	95.84	98.59	98.97	96.38	95.89	98.95	98.33	98.56	98.55	98.94	98.96	98.71	96.39	98.64	98.71	91.20	91.17	91.07	79.26	78.88	84.98
131002	95.73	95.76	95.78	97.84	95.75	95.86	95.80	97.83	95.90	100.00	97.69	97.70	95.88	95.79	98.13	97.67	95.78	95.68	95.79	95.73	95.77	95.78	95.80	97.82	95.81	95.84	91.26	91.21	91.28	79.23	79.24	85.00
LSPQ-04227	95.80	95.83	95.77	98.02	95.82	95.91	95.82	98.10	95.85	97.71	100.00	99.96	95.90	95.80	97.92	99.82	95.83	95.77	95.84	95.84	95.78	95.74	95.90	98.03	95.87	95.90	91.39	91.26	91.24	78.95	79.09	85.11
LSPQ-04228	95.80	95.74	95.77	98.04	95.81	95.85	95.82	98.06	95.85	97.69	99.96	100.00	95.90	95.80	97.91	99.85	95.80	95.72	95.77	95.84	95.76	95.76	95.88	98.05	95.88	95.87	91.28	91.24	91.25	79.05	79.22	85.04
131001	98.44	98.56	98.74	96.34	98.60	99.99	98.49	96.39	98.59	95.87	95.90	95.85	100.00	98.72	96.33	95.87	98.70	98.33	98.53	98.59	98.77	98.76	98.58	96.30	98.62	98.68	91.21	91.13	91.09	79.06	79.07	85.02
04-3911	98.52	98.90	99.87	96.23	98.99	98.66	98.66	96.34	98.92	95.79	95.77	95.78	98.71	100.00	96.34	95.94	99.91	98.37	98.49	98.58	99.87	99.89	98.65	96.23	98.62	98.65	91.13	91.02	91.03	79.46	79.17	85.19
03-8664	96.00	95.99	96.08	98.22	96.06	96.16	96.03	98.22	96.21	97.85	97.71	97.78	96.14	96.06	100.00	97.66	96.07	95.84	96.02	96.05	96.08	96.14	96.09	98.20	96.09	96.13	91.24	91.10	91.18	79.47	79.01	85.17
04-7514	95.83	95.77	95.98	97.93	95.73	95.83	95.78	98.06	95.89	97.70	99.83	99.83	95.80	95.95	97.94	100.00	95.93	95.66	95.72	95.80	95.97	95.96	95.84	97.94	95.86	95.88	91.38	91.30	91.30	79.34	79.01	85.12
KZN-2016-48390	98.54	98.87	99.84	96.33	98.94	98.70	98.60	96.33	98.92	95.77	95.79	95.79	98.69	99.95	96.32	95.95	100.00	98.29	98.56	98.62	99.83	99.84	98.67	96.26	98.66	98.69	91.03	91.06	91.09	79.51	79.21	84.98
BR-AD 2649	98.80	98.46	98.32	96.26	98.31	98.32	98.45	96.24	98.44	95.59	95.74	95.74	98.32	98.37	96.18	95.68	98.28	100.00	98.46	98.68	98.36	98.34	98.53	96.12	98.51	98.52	91.13	91.14	91.11	79.45	79.19	85.00
2590	98.61	98.62	98.52	96.27	98.49	98.57	98.48	96.32	98.56	95.76	95.81	95.81	98.49	98.55	96.27	95.78	98.57	98.44	100.00	98.85	98.52	98.53	98.80	96.25	98.75	98.77	91.00	91.03	91.01	79.01	79.08	84.94
4940	98.86	98.67	98.59	96.35	98.58	98.53	98.67	96.36	98.55	95.73	95.86	95.86	98.58	98.59	96.27	95.87	98.57	98.66	98.80	100.00	98.58	98.57	98.75	96.39	98.75	98.75	91.13	91.11	91.16	79.28	79.17	85.03
211	98.55	98.88	100.00	96.34	98.91	98.75	98.62	96.34	98.93	95.79	95.76	95.75	98.72	99.92	96.34	96.01	99.84	98.29	98.54	98.61	100.00	99.99	98.66	96.23	98.63	98.65	91.11	91.13	91.06	79.41	79.24	85.02
FH2016-1	98.54	98.87	99.98	96.36	98.88	98.75	98.61	96.32	98.96	95.79	95.77	95.79	98.72	99.92	96.33	96.00	99.83	98.29	98.55	98.63	99.98	100.00	98.69	96.20	98.62	98.65	91.07	91.07	91.06	79.38	79.24	85.04
NCTC7910	98.68	98.63	98.60	96.37	98.73	98.60	98.72	96.38	98.70	95.83	95.87	95.87	98.65	98.70	96.33	95.91	98.64	98.52	98.78	98.77	98.59	98.62	100.00	96.36	99.99	99.99	91.16	91.07	91.09	79.24	79.25	85.01
NCTC8666	96.35	96.23	96.24	99.85	96.23	96.31	96.33	99.97	96.38	97.83	98.05	98.04	96.28	96.27	98.48	97.96	96.22	96.14	96.29	96.36	96.21	96.24	96.38	100.00	96.36	96.39	91.16	91.16	91.22	79.35	79.36	85.02
NCTC7908	98.67	98.64	98.59	96.38	98.72	98.60	98.71	96.41	98.69	95.81	95.88	95.90	98.64	98.68	96.35	95.91	98.69	98.53	98.78	98.74	98.59	98.60	99.98	96.37	100.00	99.99	91.23	91.09	91.04	79.21	79.12	85.02
NCTC8639	98.69	98.66	98.64	96.34	98.69	98.67	98.70	96.44	98.68	95.81	95.85	95.87	98.63	98.69	96.33	95.88	98.69	98.51	98.77	98.76	98.64	98.65	99.99	96.39	99.98	100.00	91.12	91.05	91.11	79.23	79.23	85.08
PO100/5	91.11	91.11	91.07	91.20	91.03	91.09	90.99	91.24	91.19	91.26	91.29	91.30	91.17	91.08	91.39	91.39	91.03	91.15	91.01	91.07	91.06	91.07	91.10	91.16	91.12	91.10	100.00	99.77	99.77	79.41	78.93	85.69
KL1196	91.01	91.07	91.07	91.23	91.00	91.13	91.00	91.24	91.17	91.18	91.35	91.32	91.13	91.04	91.32	91.34	91.06	91.09	91.06	91.06	91.09	91.09	91.07	91.11	91.06	91.06	99.75	100.00	99.98	79.49	78.88	85.63
W25	91.06	91.06	91.04	91.18	91.05	91.07	91.02	91.21	91.15	91.30	91.27	91.28	91.07	91.11	91.37	91.35	91.04	91.11	91.03	91.05	91.05	91.00	91.08	91.12	91.11	91.04	99.77	100.00	100.00	79.41	78.88	85.62
C. belfanti FRC0043T	79.38	79.42	79.33	79.38	79.44	79.14	79.26	79.31	79.50	79.09	79.11	79.07	79.12	79.38	79.35	79.30	79.47	79.56	79.16	79.51	79.42	79.30	79.28	79.06	79.43	79.40	79.46	79.42	79.61	100.00	95.10	78.53
C. diphtheriae DSM 44123T	79.26	79.33	79.53	79.24	79.55	79.38	79.05	79.29	79.37	79.31	79.05	79.21	79.40	79.17	78.83	79.06	79.45	79.70	79.04	79.57	79.48	79.46	79.18	79.20	79.42	79.37	79.16	79.09	79.17	95.22	100.00	78.57
C. pseudotuberculosis DSM 2068	84.90	84.97	85.08	85.06	85.20	85.05	84.99	85.08	85.12	85.05	84.93	84.92	84.98	85.11	85.11	85.15	84.82	84.86	85.00	84.85	85.05	85.01	84.98	85.04	84.90	85.10	85.62	85.52	85.50	78.40	78.42	100.00
