# Supplementary material for: Phylogenomic characterisation of a novel corynebacterial species pathogenic to animals
Source: Antonie Van Leeuwenhoek. 2020 Jun 4;113(8):1225–39. doi: 10.1007/s10482-020-01430-5 (PMC7334274; doi:10.1007/s10482-020-01430-5)
Supplement: Supplementary file 4 — Supplementary material 4 (PDF 207 kb) [file 10482_2020_1430_MOESM4_ESM.pdf]

Supplementary Fig. 1. A multiple sequence alignment of the *tox* genes plus 100 bp upstream and downstream of the gene from *C. ulcerans* 102, *C. diphtheriae* NCTC 13129 and the tree strains belonging to the novel species using CLUSTAL O (1.2.4). The promotor region, start codon and the two base-pair (GG) insertion in strains W25 and KL1196 are highlighted in yellow.

```
C.ulcerans_0102             tgtaaccaatctatcaaaaaatgttattgatt-ccgggccaccctataattaggatagct      59
C.diphtheriae_NCTC13129     tgtaaccaatctatcaaaaaagggcattgatttcagagcacccttataattaggatagct      60
C.ulcerans_KL1196           tgtaaccaatctatcaaaaaatgttattgatttcggaccacccctctaattaggatagat      60
C.ulcerans_W25              tgtaaccaatctatcaaaaaatgttattgatttcggaccacccctctaattaggatagat      60
C.ulcerans_PO100/5          tgtaaccaatctatcaaaaaatgttattgatttcggaccacccctataattaggataggt      60
                            ********************* *  ******* * *  *  ** * ************ *

                              -10 promotor region                  +1 translation start
C.ulcerans_0102             ttacctaattattttatgagtcctggtaaggggatgcgttgtgaacagaaaactgtttgc      119
C.diphtheriae_NCTC13129     ttacctaattattttatgagtcctggtaaggggatacgttgtgagcagaaaactgtttgc      120
C.ulcerans_KL1196           ttatctaattattttatgagtcctggtaaggggatacgttgtgaacagaaaactgtttgc      120
C.ulcerans_W25              ttatctaattattttatgagtcctggtaaggggatacgttgtgaacagaaaactgtttgc      120
C.ulcerans_PO100/5          ttatctaattattttatgagtcctggtaaggggatacgttgtgaacagaaaactgtttgc      120
                            *** ******************************* ******** ***************

                                                      2 bp insertion
C.ulcerans_0102             gtcaatcttgataggggcgctactgggg--ataggggccccaccttcagcccatgcaggc      177
C.diphtheriae_NCTC13129     gtcaatcttaataggggcgctactgggg--ataggggccccaccttcagcccatgcaggc      178
C.ulcerans_KL1196           gtcaatcttaataggggcgctgctggggggatagggaccccactttcagctcatgcaagc      180
C.ulcerans_W25              gtcaatcttaataggggcgctgctggggggatagggaccccactttcagctcatgcaagc      180
C.ulcerans_PO100/5          gtcaatcttaataggggcgctgctgggg--atagggaccccactttcagctcatgcaagc      178
                            ********* *********** *****   ****** ****** ****** ****** **

C.ulcerans_0102             gctgatgatgttgttgattcttctaaatcttttgtgatggaaaacttttcttcgtatcac      237
C.diphtheriae_NCTC13129     gctgatgatgttgttgattcttctaaatcttttgtgatggaaaacttttcttcgtaccac      238
C.ulcerans_KL1196           gctgatgatgttgttgattcttctaaatcttttgtgatggaaaacttttcttcgtaccac      240
C.ulcerans_W25              gctgatgatgttgttgattcttctaaatcttttgtgatggaaaacttttcttcgtaccac      240
C.ulcerans_PO100/5          gctgatgatgttgttgattcttctaaatcttttgtgatggaaaacttttcttcgtaccac      238
                            ******************************************************** ***

C.ulcerans_0102             gggactaagcctgggtatgtagattccattcaaaaaggtatacaaaagccaaaatctggt      297
C.diphtheriae_NCTC13129     gggactaaacctggttatgtagattccattcaaaaaggtatacaaaagccaaaatctggt      298
C.ulcerans_KL1196           gggactaaacctgggtatgtagattccattcaaaaaggtatacaaaagccaaaatctggt      300
C.ulcerans_W25              gggactaaacctgggtatgtagattccattcaaaaaggtatacaaaagccaaaatctggt      300
C.ulcerans_PO100/5          gggactaaacctgggtatgtagattccattcaaaaaggtatacaaaagccaaaatctggt      298
                            ******** ***** *********************************************

C.ulcerans_0102             gcacaaggaaattatgacgatgattggaaaggattttatagtaccgacaataaatacgac      357
C.diphtheriae_NCTC13129     acacaaggaaattatgacgatgattggaaagggttttatagtaccgacaataaatacgac      358
C.ulcerans_KL1196           acgcaaggaaattatgacgatgattggaaagggttttatagtaccgacaataaatacgac      360
C.ulcerans_W25              acgcaaggaaattatgacgatgattggaaagggttttatagtaccgacaataaatacgac      360
C.ulcerans_PO100/5          acgcaaggaaattatgacgatgattggaaagggttttatagtaccgacaataaatacgac      358
                             * ***************************** ***************************

C.ulcerans_0102             gctgcgggatactctgtagataatgaaaacccgctgtctggaaaggctggaggcgtggtc      417
C.diphtheriae_NCTC13129     gctgcgggatactctgtagataatgaaaacccgctctctggaaaagctggaggcgtggtc      418
C.ulcerans_KL1196           gctgcgggatactctgtagataatgaaaacccgctctctggaaaagctggaggcgtggtc      420
C.ulcerans_W25              gctgcgggatactctgtagataatgaaaacccgctctctggaaaagctggaggcgtggtc      420
C.ulcerans_PO100/5          gctgcgggatactctgtagataatgaaaacccgctctctggaaaagctggaggcgtggtc      418
                            *********************************** ******** ***************

C.ulcerans_0102             aaagtgacgtatccaggactgacaaagattctcgccctaaaggtggataatgccgaaact      477
C.diphtheriae_NCTC13129     aaagtgacgtatccaggactgacgaaggttctcgcactaaaagtggataatgccgaaact      478
C.ulcerans_KL1196           aaagtgacgtatccaggactgacgaagattctcgcgctaaaagtggataatgccgaaact      480
C.ulcerans_W25              aaagtgacgtatccaggactgacgaagattctcgcgctaaaagtggataatgccgaaact      480
C.ulcerans_PO100/5          aaagtgacgtatccaggactgacgaagattctcgcgctaaaagtggataatgccgaaact      478
                            *********************** *** ******* ***** ******************

C.ulcerans_0102             attaagaaagagttaggtctaagtctcactgaaccgctgatggagcaagtcggaacggaa      537
C.diphtheriae_NCTC13129     attaagaaagagttaggtttaagtctcactgaaccgttgatggagcaagtcggaacggaa      538
C.ulcerans_KL1196           attaagaaagagttaggtctaagtctcactgaaccactgatggagcaagtcggatcggaa      540
C.ulcerans_W25              attaagaaagagttaggtctaagtctcactgaaccactgatggagcaagtcggatcggaa      540
C.ulcerans_PO100/5          attaagaaagagttaggtctaagtctcactgaaccactgatggagcaagtcggatcggaa      538
                            ****************** ****************  ***************** *****

C.ulcerans_0102             gagtttatcaaaaggttcggtgatggtgcttcgcgtgtagtgctcagccttcccttcgct      597
C.diphtheriae_NCTC13129     gagtttatcaaaaggttcggtgatggtgcttcgcgtgtagtgctcagccttcccttcgct      598
C.ulcerans_KL1196           gagtttatcaaaaggtttggtgatggtgcttcacgtgtagtactcagccttccctttgct      600
C.ulcerans_W25              gagtttatcaaaaggtttggtgatggtgcttcacgtgtagtactcagccttccctttgct      600
C.ulcerans_PO100/5          gagtttatcaaaaggtttggtgatggtgcttcacgtgtagtactcagccttccctttgct      598
                            ***************** ************** ******** ************** ***
```

```
C.ulcerans_0102               gaggggagttctagtgttgaatatattaataactgggaacaggcgaaagagttaagcgta      657
C.diphtheriae_NCTC13129       gaggggagttctagcgttgaatatattaataactgggaacaggcgaaagcgttaagcgta      658
C.ulcerans_KL1196             gaggggagttctagcgttgaatatattaataactgggaacaggcgaaagcgttaagcgta      660
C.ulcerans_W25                gaggggagttctagcgttgaatatattaataactgggaacaggcgaaagcgttaagcgta      660
C.ulcerans_PO100/5            gaggggagttctagcgttgaatatattaataactgggaacaggcgaaagcgttaagcgta      658
                              ************** ********************************** **********

C.ulcerans_0102               gaacttgagattaattttgaaacccgtggaaaacgtggccaagatgcgatgtatgagtat      717
C.diphtheriae_NCTC13129       gaacttgagattaattttgaaacccgtggaaaacgtggccaagatgcgatgtatgagtat      718
C.ulcerans_KL1196             gaacttgagattaattttgaaacccgtggaaaacgtggccaagatgcgatgtatgagtat      720
C.ulcerans_W25                gaacttgagattaattttgaaacccgtggaaaacgtggccaagatgcgatgtatgagtat      720
C.ulcerans_PO100/5            gaacttgagattaattttgaaacccgtggaaaacgtggccaagatgcgatgtatgagtat      718
                              ************************************************************

C.ulcerans_0102               atggctcaatcgtgtgcgggaaatcgtgtcaggcgatcagtgggtagctcattgtcatgc      777
C.diphtheriae_NCTC13129       atggctcaagcctgtgcaggaaatcgtgtcaggcgatcagtaggtagctcattgtcatgc      778
C.ulcerans_KL1196             atggctcaatcgtgtgcgggaaatcgtgtcaggcgatcagtaggtaattcatcgtcatgc      780
C.ulcerans_W25                atggctcaatcgtgtgcgggaaatcgtgtcaggcgatcagtaggtaattcatcgtcatgc      780
C.ulcerans_PO100/5            atggctcaatcgtgtgcgggaaatcgtgtcaggcgatcagtaggtaattcatcgtcatgc      778
                              ********* * ***** *********************** ****  **** *******

C.ulcerans_0102               ataaatcttgattgggatgccataagggataaaactaagacaaagatagagtctttgaaa      837
C.diphtheriae_NCTC13129       ataaatcttgattgggatgtcataagggataaaactaagacaaagatagagtctttgaaa      838
C.ulcerans_KL1196             ataaatcttgattgggatgctataagggataaaactaagacaaagatagagtctttaaaa      840
C.ulcerans_W25                ataaatcttgattgggatgctataagggataaaactaagacaaagatagagtctttaaaa      840
C.ulcerans_PO100/5            ataaatcttgattgggatgctataagggataaaactaagacaaagatagagtctttaaaa      838
                              *******************  *********************************** ***

C.ulcerans_0102               gagcatggccctatcaaaaataaaatgagcgaaagtcccaataaaacagtctctgaggaa      897
C.diphtheriae_NCTC13129       gagcatggccctatcaaaaataaaatgagcgaaagtcccaataaaacagtatctgaggaa      898
C.ulcerans_KL1196             gagcatggtcctatcaaaaataaaatgagcgaaagtcccaataaagcggtatctgaggaa      900
C.ulcerans_W25                gagcatggtcctatcaaaaataaaatgagcgaaagtcccaataaagcggtatctgaggaa      900
C.ulcerans_PO100/5            gagcatggtcctatcaaaaataaaatgagcgaaagtcccaataaagcggtatctgaggaa      898
                              ******** ************************************ * ** *********

C.ulcerans_0102               aaggctaaacaatatctagaagaatttcatcaaacggcattagagcatcctgaattgtca      957
C.diphtheriae_NCTC13129       aaagctaaacaatacctagaagaatttcatcaaacggcattagagcatcctgaattgtca      958
C.ulcerans_KL1196             aaagctaaacaatatctagaagaatttcatcaaacggcattagaacatcctgaattgtca      960
C.ulcerans_W25                aaagctaaacaatatctagaagaatttcatcaaacggcattagaacatcctgaattgtca      960
C.ulcerans_PO100/5            aaagctaaacaatatctagaagaatttcatcaaacggcattagaacatcctgaattgtca      958
                              ** *********** ***************************** ***************

C.ulcerans_0102               gaacttaaaaccgttactggggtcaattctgtatttgctggggctaactatgcgtcgtgg      1017
C.diphtheriae_NCTC13129       gaacttaaaaccgttactgggaccaatcctgtattcgctggggctaactatgcggcgtgg      1018
C.ulcerans_KL1196             gaacttaaaaccgttactgggaccaattctgtattcgctggggctaactatgcggcgtgg      1020
C.ulcerans_W25                gaacttaaaaccgttactgggaccaattctgtattcgctggggctaactatgcggcgtgg      1020
C.ulcerans_PO100/5            gaacttaaaaccgttactgggaccaattctgtattcgctggggctaactatgcggcgtgg      1018
                              *********************  **** ******* ****************** *****

C.ulcerans_0102               gcggtaaacgttgcgcaagttgtcgatagtaaaacagctgataatttagaaaagacaact      1077
C.diphtheriae_NCTC13129       gcagtaaacgttgcgcaagttatcgatagcgaaacagctgataatttggaaaagacaact      1078
C.ulcerans_KL1196             gcagtaaacgttgcgcaagttatcgatagcgaaacagctgataatttggaaaagacaact      1080
C.ulcerans_W25                gcagtaaacgttgcgcaagttatcgatagcgaaacagctgataatttggaaaagacaact      1080
C.ulcerans_PO100/5            gcagtaaacgttgcgcaagttatcgatagcgaaacagctgataatttggaaaagacaact      1078
                              ** ****************** *******  **************** ************

C.ulcerans_0102               gctgctctttcgatacttcctggtatcggtagcgtaatgggtattgcagacggtgccgtt      1137
C.diphtheriae_NCTC13129       gctgctctttcgatacttcctggtatcggtagcgtaatgggcattgcagacggtgccgtt      1138
C.ulcerans_KL1196             gctgctctttcgatacttcctggtatcggtagcgtaatgggtattgcggacggtgccgtt      1140
C.ulcerans_W25                gctgctctttcgatacttcctggtatcggtagcgtaatgggtattgcggacggtgccgtt      1140
C.ulcerans_PO100/5            gctgctctttcgatacttcctggtatcggtagcgtaatgggtattgcggacggtgccgtt      1138
                              ***************************************** ***** ************

C.ulcerans_0102               caccacaatacggaagagatagtggcacaatcgatagctttatcgtctttaatggttgct      1197
C.diphtheriae_NCTC13129       caccacaatacagaagagatagtggcacaatcaatagctttatcgtctttaatggttgct      1198
C.ulcerans_KL1196             caccacaatactgaagagatagtggcacaatcaatagctttatcgtctttaatggttgct      1200
C.ulcerans_W25                caccacaatactgaagagatagtggcacaatcaatagctttatcgtctttaatggttgct      1200
C.ulcerans_PO100/5            caccacaatactgaagagatagtggcacaatcaatagctttatcgtctttaatggttgct      1198
                              *********** ******************** ***************************

C.ulcerans_0102               caagctataccattggtaggagagctagttgatcttggttttgctgcatataattttgta      1257
C.diphtheriae_NCTC13129       caagctattccattggtaggagagctagttgatattggtttcgctgcatataattttgta      1258
C.ulcerans_KL1196             caagctataccattggtaggagagctagttgatattggtttcgctgcatataattttgta      1260
```

```
C.ulcerans_W25                caagctataccattggtaggagagctagttgatattggtttcgctgcatataattttgta    1260
C.ulcerans_PO100/5            caagctataccattggtaggagagctagttgatattggtttcgctgcatataattttgta    1258
                              ******** ************************ ******* ******************

C.ulcerans_0102               gagagtattatcaatttatttcaagtagttcataattcgtataatcgtcccgcgtactct    1317
C.diphtheriae_NCTC13129       gagagtattatcaatttatttcaagtagttcataattcgtataatcgtcccgcgtattct    1318
C.ulcerans_KL1196             gagagtattatcaatttatttcaagtagttcataattcgtataatcgtcccgcgtattct    1320
C.ulcerans_W25                gagagtattatcaatttatttcaagtagttcataattcgtataatcgtcccgcgtattct    1320
C.ulcerans_PO100/5            gagagtattatcaatttatttcaagtagttcataattcgtataatcgtcccgcgtattct    1318
                              ******************************************************** ***

C.ulcerans_0102               ccggggcataaaacgcaaccatttgttcatggcggatatgctgccagttggaacactgtt    1377
C.diphtheriae_NCTC13129       ccggggcataaaacacaaccatttcttcatgacgggtatgctgtcagttggaacactgtt    1378
C.ulcerans_KL1196             ccggggcataaaacgcaaccatttcttcatgacgggtatgctgtcagttggaacactgtt    1380
C.ulcerans_W25                ccggggcataaaacgcaaccatttcttcatgacgggtatgctgtcagttggaacactgtt    1380
C.ulcerans_PO100/5            ccggggcataaaacgcaaccatttcttcatgacgggtatgctgtcagttggaacactgtt    1378
                              ************** ********* ****** *** ******* ****************

C.ulcerans_0102               gaagattcgataatcaaaactggttttcaaggcgagagcgggcacgacataaaaattact    1437
C.diphtheriae_NCTC13129       gaagattcgataatccgaactggttttcaaggggagagtgggcacgacataaaaattact    1438
C.ulcerans_KL1196             gaagattcgataatccaaactggttttcaaggggagagtgggcacgacataaaaattact    1440
C.ulcerans_W25                gaagattcgataatccaaactggttttcaaggggagagtgggcacgacataaaaattact    1440
C.ulcerans_PO100/5            gaagattcgataatccaaactggttttcaaggggagagtgggcacgacataaaaattact    1438
                              ***************  *************** ***** *********************

C.ulcerans_0102               gctgaaaataccccgcttccaatcgcgggtgtcctactaccgactattcctggaaagctg    1497
C.diphtheriae_NCTC13129       gctgaaaataccccgcttccaatcgcgggtgtcctactaccgactattcctggaaagctg    1498
C.ulcerans_KL1196             gctgaaaataccccgcttccaatcgcgggtgtcctactaccgactattcctggaaagctg    1500
C.ulcerans_W25                gctgaaaataccccgcttccaatcgcgggtgtcctactaccgactattcctggaaagctg    1500
C.ulcerans_PO100/5            gctgaaaataccccgcttccaatcgcgggtgtcctactaccgactattcctggaaagctg    1498
                              ************************************************************

C.ulcerans_0102               gacgttaataagtccaagactcatatttccgtaaatggtcgaaaaataaggatgcgatgc    1557
C.diphtheriae_NCTC13129       gacgttaataagtccaagactcatatttccgtaaatggtcggaaaataaggatgcgttgc    1558
C.ulcerans_KL1196             gacgttaataagtccaagactcatatttccgtaaatggtcggaaaataaggatgcgatgc    1560
C.ulcerans_W25                gacgttaataagtccaagactcatatttccgtaaatggtcggaaaataaggatgcgatgc    1560
C.ulcerans_PO100/5            gacgttaataagtccaagactcatatttccgtaaatggtcggaaaataaggatgcgatgc    1558
                              ***************************************** ************** ***

C.ulcerans_0102               agagctatagatgatgctacaaccttttgccgccctaaaacccctgtttatgttggtaat    1617
C.diphtheriae_NCTC13129       agagctatagacggtgatgtaactttttgtcgccctaaatctcctgtttatgttggtaat    1618
C.ulcerans_KL1196             agagctatagacggtgatgtaaccttttgtcgccctaaatctcctgtttatgttggtaat    1620
C.ulcerans_W25                agagctatagacggtgatgtaaccttttgtcgccctaaatctcctgtttatgttggtaat    1620
C.ulcerans_PO100/5            agagctatagacggtgatgtaaccttttgtcgccctaaatctcctgtttatgttggtaat    1618
                              *********** * ** *  *** ***** ********* * ******************

C.ulcerans_0102               ggggtgcatgcgaatcttcatgtggcatttcatagaagtagctctgagaaaattcattct    1677
C.diphtheriae_NCTC13129       ggtgtgcatgcgaatcttcacgtggcatttcacagaagcagctcggagaaaattcattct    1678
C.ulcerans_KL1196             ggtgtgcatgcgaatcttcacgtggcatttcatagaagcagctcggagaaaattcattct    1680
C.ulcerans_W25                ggtgtgcatgcgaatcttcacgtggcatttcatagaagcagctcggagaaaattcattct    1680
C.ulcerans_PO100/5            ggtgtgcatgcgaatcttcacgtggcatttcatagaagcagctcggagaaaattcattct    1678
                              ** ***************** *********** ***** ***** ***************

C.ulcerans_0102               gatgaaactccgttgagctccatagacgtgcttgggtaccagaaaacagtggatcacacc    1737
C.diphtheriae_NCTC13129       aatgaaatttcgtcggattccataggcgttcttgggtaccagaaaacagtagatcacacc    1738
C.ulcerans_KL1196             aatgaaatttcgtcggattccataggcgttcttggataccagaaaatagtagatcacacc    1740
C.ulcerans_W25                aatgaaatttcgtcggattccataggcgttcttggataccagaaaatagtagatcacacc    1740
C.ulcerans_PO100/5            aatgaaatttcgtcggattccataggcgttcttggataccagaaaatagtagatcacacc    1738
                               ****** * *** *   ******* *** ***** ********** *** *********

C.ulcerans_0102               aaagtgaattctaagctatcgctatttgctgaagtcaaaagctgaaaagttagtggggtc    1797
C.diphtheriae_NCTC13129       aaggttaattctaagctatcgctattttttgaaatcaaaagctgaaa-ggtagtggggtc    1797
C.ulcerans_KL1196             aaggttaattctaagctatcgctattttttgaaatcaaaagctgaaa-ggtagtggggtc    1799
C.ulcerans_W25                aaggttaattctaagctatcgctattttttgaaatcaaaagctgaaa-ggtagtggggtc    1799
C.ulcerans_PO100/5            aaggttaattctaagctatcgctattttttgaaatcaaaagctgaaa-ggtagtggggtc    1797
                              ** ** *********************  **** ************* * **********

C.ulcerans_0102               gtgtgccggtaagccgaacaggtccggaatggtattggagtaatccaaggtaaaaggcta    1857
C.diphtheriae_NCTC13129       gtgtgccggtaagccgaacggttccggaatggcgctatagtatgcacaggtagagcagaa    1857
C.ulcerans_KL1196             gtgtgccggtaagccgaacggttccggaatggcgctatagtatgcacagttagagcagaa    1859
C.ulcerans_W25                gtgtgccggtaagccgaacggttccggaatggcgctatagtatgcacagttagagcagaa    1859
C.ulcerans_PO100/5            gtgtgccggtaagccgaacggttccggaatggcgctatagtatgcacagttagagcagaa    1857
                              ****************** * **********   *  ****  *  ** ** *     *
```

```
C.ulcerans_0102             ttagcatctgactacggatcagaagg 1883
C.diphtheriae_NCTC13129     ttcgaatctgactacggatcagaagg 1883
C.ulcerans_KL1196           ttcttatctgactacggatcagaagg 1885
C.ulcerans_W25              ttcttatctgactacggatcagaagg 1885
C.ulcerans_PO100/5          ttcttatctgactacggatcagaagg 1883
                            **   *********************
```
