# Supplementary material for: Phylogenomic characterisation of a novel corynebacterial species pathogenic to animals
Source: Antonie Van Leeuwenhoek. 2020 Jun 4;113(8):1225–39. doi: 10.1007/s10482-020-01430-5 (PMC7334274; doi:10.1007/s10482-020-01430-5)
Supplement: Supplementary file 5 — Supplementary material 5 (DOCX 13 kb) [file 10482_2020_1430_MOESM5_ESM.docx]

**Supplementary Table 4.** List of genes conserved only among *C. ulcerans* strains (based on genome comparison using Roary with 70% sequence identity)

| ***C. ulcerans***  **NCTC 7910** | **Function** |
| --- | --- |
| cp25_00182 | hypothetical protein |
| cp25_00236 | hypothetical protein |
| cp25_00398 | hypothetical protein |
| cp25_00696 | Heat-inducible transcription repressor HrcA |
| cp25_00703 | hypothetical protein |
| cp25_00774 | Aminopeptidase N |
| cp25_00837 | hypothetical protein |
| cp25_00876 | Putative fluoride ion transporter CrcB |
| cp25_00937 | hypothetical protein |
| cp25_00950 | Cysteine--tRNA ligase |
| cp25_00955 | hypothetical protein |
| cp25_00967 | hypothetical protein |
| cp25_00968 | hypothetical protein |
| cp25_00969 | hypothetical protein |
| cp25_01068 | hypothetical protein |
| cp25_01114 | hypothetical protein |
| cp25_01156 | putative propionyl-CoA carboxylase beta chain 5 |
| cp25_01274 | hypothetical protein |
| cp25_01350 | hypothetical protein |
| cp25_01401 | hypothetical protein |
| cp25_01454 | hypothetical protein |
| cp25_01498 | hypothetical protein |
| cp25_01632 | hypothetical protein |
| cp25_01941 | hypothetical protein |
| cp25_01949 | hypothetical protein |
| cp25_02069 | RNA polymerase sigma factor YlaC |
| cp25_02074 | 1,4-dihydroxy-2-naphthoyl-CoA synthase |
| cp25_02076 | hypothetical protein |
| cp25_02094 | hypothetical protein |
| cp25_02113 | hypothetical protein |
