# Supplementary material for: Phylogenomic characterisation of a novel corynebacterial species pathogenic to animals
Source: Antonie Van Leeuwenhoek. 2020 Jun 4;113(8):1225–39. doi: 10.1007/s10482-020-01430-5 (PMC7334274; doi:10.1007/s10482-020-01430-5)
Supplement: Supplementary file 6 — Supplementary material 6 (DOCX 22 kb) [file 10482_2020_1430_MOESM6_ESM.docx]

**Supplementary Table 5.** List of genes specific to the clade containing strains W25, PO100/5 and KL1196 (based on genome comparison using Roary with 70% sequence identity).

| **W25** | **PO100/5** | **KL1196** | **Annotation** |
| --- | --- | --- | --- |
| cp29_00014 | cp18_02462 | cp22_02560 | hypothetical protein |
| cp29_00031 | cp18_02443 | cp22_02543 | hypothetical protein |
| cp29_00036 | cp18_02438 | cp22_02538 | hypothetical protein |
| cp29_00060 | cp18_02411 | cp22_02514 | hypothetical protein |
| cp29_00077 | cp18_02394 | cp22_02497 | hypothetical protein |
| cp29_00097 | cp18_02374 | cp22_00913 | UDP-glucose 6-dehydrogenase |
| cp29_00140 | cp18_02332 | cp22_02401 | hypothetical protein |
| cp29_00148 | cp18_02324 | cp22_02393 | putative peptidase |
| cp29_00161 | cp18_02312 | cp22_02380 | Transcriptional regulatory protein DesR |
| cp29_00162 | cp18_02311 | cp22_02379 | hypothetical protein |
| cp29_00163 | cp18_02310 | cp22_02378 | hypothetical protein |
| cp29_00164 | cp18_02309 | cp22_02377 | hypothetical protein |
| cp29_00165 | cp18_02308 | cp22_02376 | Daunorubicin/doxorubicin resistance ATP-binding protein DrrA |
| cp29_00166 | cp18_02307 | cp22_02375 | hypothetical protein |
| cp29_00169 | cp18_02304 | cp22_02372 | hypothetical protein |
| cp29_00171 | cp18_02301 | cp22_02370 | hypothetical protein |
| cp29_00172 | cp18_02300 | cp22_02369 | hypothetical protein |
| cp29_00173 | cp18_02299 | cp22_02368 | hypothetical protein |
| cp29_00174 | cp18_02297 | cp22_02367 | hypothetical protein |
| cp29_00175 | cp18_02295 | cp22_02366 | hypothetical protein |
| cp29_00187 | cp18_02283 | cp22_02354 | hypothetical protein |
| cp29_00198 | cp18_02272 | cp22_02343 | hypothetical protein |
| cp29_00199 | cp18_02271 | cp22_02342 | hypothetical protein |
| cp29_00200 | cp18_02270 | cp22_02341 | hypothetical protein |
| cp29_00204 | cp18_02266 | cp22_02337 | hypothetical protein |
| cp29_00205 | cp18_02265 | cp22_02336 | hypothetical protein |
| cp29_00214 | cp18_02255 | cp22_02327 | hypothetical protein |
| cp29_00238 | cp18_02230 | cp22_02303 | hypothetical protein |
| cp29_00239 | cp18_02229 | cp22_02302 | hypothetical protein |
| cp29_00250 | cp18_02219 | cp22_00633 | UDP-3-O-(3-hydroxymyristoyl)glucosamine N-acyltransferase |
| cp29_00252 | cp18_02217 | cp22_00635 | hypothetical protein |
| cp29_00253 | cp18_02216 | cp22_00636 | hypothetical protein |
| cp29_00281 | cp18_02188 | cp22_00664 | hypothetical protein |
| cp29_00291 | cp18_02178 | cp22_00674 | hypothetical protein |
| cp29_00307 | cp18_02161 | cp22_00690 | hypothetical protein |
| cp29_00308 | cp18_02160 | cp22_00691 | hypothetical protein |
| cp29_00315 | cp18_02151 | cp22_00696 | hypothetical protein |
| cp29_00316 | cp18_02150 | cp22_00697 | hypothetical protein |
| cp29_00318 | cp18_02148 | cp22_00699 | hypothetical protein |
| cp29_00319 | cp18_02147 | cp22_00700 | hypothetical protein |
| cp29_00320 | cp18_02146 | cp22_00701 | hypothetical protein |
| cp29_00321 | cp18_02145 | cp22_00702 | hypothetical protein |
| cp29_00322 | cp18_02144 | cp22_00703 | hypothetical protein |
| cp29_00323 | cp18_02143 | cp22_00704 | hypothetical protein |
| cp29_00324 | cp18_02142 | cp22_00705 | hypothetical protein |
| cp29_00325 | cp18_02141 | cp22_00706 | hypothetical protein |
| cp29_00326 | cp18_02140 | cp22_00707 | hypothetical protein |
| cp29_00327 | cp18_02139 | cp22_00708 | hypothetical protein |
| cp29_00328 | cp18_02138 | cp22_00709 | hypothetical protein |
| cp29_00329 | cp18_02137 | cp22_00710 | hypothetical protein |
| cp29_00330 | cp18_02136 | cp22_00711 | hypothetical protein |
| cp29_00331 | cp18_02135 | cp22_00712 | hypothetical protein |
| cp29_00332 | cp18_02134 | cp22_00713 | hypothetical protein |
| cp29_00333 | cp18_02133 | cp22_00714 | hypothetical protein |
| cp29_00335 | cp18_02126 | cp22_00870 | hypothetical protein |
| cp29_00413 | cp18_00726 | cp22_00794 | hypothetical protein |
| cp29_00414 | cp18_00727 | cp22_00793 | hypothetical protein |
| cp29_00415 | cp18_00728 | cp22_00792 | hypothetical protein |
| cp29_00416 | cp18_00729 | cp22_00791 | hypothetical protein |
| cp29_00417 | cp18_00730 | cp22_00790 | hypothetical protein |
| cp29_00418 | cp18_00731 | cp22_00789 | hypothetical protein |
| cp29_00419 | cp18_00732 | cp22_00788 | hypothetical protein |
| cp29_00420 | cp18_00733 | cp22_00787 | hypothetical protein |
| cp29_00421 | cp18_00734 | cp22_00786 | hypothetical protein |
| cp29_00422 | cp18_00735 | cp22_00785 | hypothetical protein |
| cp29_00423 | cp18_00736 | cp22_00784 | hypothetical protein |
| cp29_00424 | cp18_00737 | cp22_00783 | hypothetical protein |
| cp29_00425 | cp18_00738 | cp22_00782 | hypothetical protein |
| cp29_00426 | cp18_00739 | cp22_00781 | hypothetical protein |
| cp29_00428 | cp18_00741 | cp22_00779 | hypothetical protein |
| cp29_00430 | cp18_00743 | cp22_00777 | hypothetical protein |
| cp29_00431 | cp18_00744 | cp22_00776 | hypothetical protein |
| cp29_00432 | cp18_00745 | cp22_00775 | hypothetical protein |
| cp29_00434 | cp18_00747 | cp22_00773 | hypothetical protein |
| cp29_00438 | cp18_00750 | cp22_00769 | hypothetical protein |
| cp29_00440 | cp18_00752 | cp22_00767 | hypothetical protein |
| cp29_00445 | cp18_00759 | cp22_00762 | hypothetical protein |
| cp29_00447 | cp18_00761 | cp22_00760 | hypothetical protein |
| cp29_00449 | cp18_00763 | cp22_00758 | hypothetical protein |
| cp29_00451 | cp18_00765 | cp22_00756 | hypothetical protein |
| cp29_00452 | cp18_00766 | cp22_00755 | hypothetical protein |
| cp29_00454 | cp18_00768 | cp22_00753 | hypothetical protein |
| cp29_00455 | cp18_00769 | cp22_00752 | hypothetical protein |
| cp29_00459 | cp18_00771 | cp22_00748 | hypothetical protein |
| cp29_00464 | cp18_00776 | cp22_00743 | hypothetical protein |
| cp29_00465 | cp18_00777 | cp22_00742 | hypothetical protein |
| cp29_00466 | cp18_00778 | cp22_00741 | hypothetical protein |
| cp29_00467 | cp18_00779 | cp22_00740 | hypothetical protein |
| cp29_00468 | cp18_00780 | cp22_00739 | hypothetical protein |
| cp29_00469 | cp18_00781 | cp22_00738 | hypothetical protein |
| cp29_00481 | cp18_02040 | cp22_00725 | hypothetical protein |
| cp29_00482 | cp18_02039 | cp22_00724 | hypothetical protein |
| cp29_00493 | cp18_02026 | cp22_02292 | hypothetical protein |
| cp29_00494 | cp18_02025 | cp22_02291 | hypothetical protein |
| cp29_00519 | cp18_02000 | cp22_02266 | hypothetical protein |
| cp29_00521 | cp18_01997 | cp22_02264 | hypothetical protein |
| cp29_00530 | cp18_01988 | cp22_02255 | hypothetical protein |
| cp29_00532 | cp18_01986 | cp22_02253 | hypothetical protein |
| cp29_00556 | cp18_01962 | cp22_02229 | hypothetical protein |
| cp29_00604 | cp18_01913 | cp22_02181 | hypothetical protein |
| cp29_00609 | cp18_01908 | cp22_02176 | hypothetical protein |
| cp29_00611 | cp18_01905 | cp22_02174 | hypothetical protein |
| cp29_00646 | cp18_01871 | cp22_00029 | hypothetical protein |
| cp29_00662 | cp18_01856 | cp22_00044 | hypothetical protein |
| cp29_00698 | cp18_01820 | cp22_00080 | hypothetical protein |
| cp29_00710 | cp18_01809 | cp22_00092 | hypothetical protein |
| cp29_00719 | cp18_01800 | cp22_00101 | hypothetical protein |
| cp29_00721 | cp18_01798 | cp22_00103 | Vitamin B12 import system permease protein BtuC |
| cp29_00724 | cp18_01795 | cp22_00106 | Hemin transport system permease protein HmuU |
| cp29_00828 | cp18_01691 | cp22_00210 | hypothetical protein |
| cp29_00838 | cp18_01681 | cp22_00220 | Ammonia channel |
| cp29_00840 | cp18_01679 | cp22_00222 | hypothetical protein |
| cp29_00855 | cp18_01663 | cp22_00237 | hypothetical protein |
| cp29_00861 | cp18_01657 | cp22_00243 | D-amino acid dehydrogenase |
| cp29_00864 | cp18_01654 | cp22_00246 | hypothetical protein |
| cp29_00865 | cp18_01653 | cp22_00247 | hypothetical protein |
| cp29_00866 | cp18_01652 | cp22_00248 | hypothetical protein |
| cp29_00867 | cp18_01651 | cp22_00249 | hypothetical protein |
| cp29_00868 | cp18_01650 | cp22_00250 | hypothetical protein |
| cp29_00869 | cp18_01649 | cp22_00251 | hypothetical protein |
| cp29_00870 | cp18_01648 | cp22_00252 | hypothetical protein |
| cp29_00871 | cp18_01647 | cp22_00253 | hypothetical protein |
| cp29_00873 | cp18_01645 | cp22_00255 | hypothetical protein |
| cp29_00874 | cp18_01644 | cp22_00256 | hypothetical protein |
| cp29_00875 | cp18_01643 | cp22_00257 | hypothetical protein |
| cp29_00876 | cp18_01642 | cp22_00258 | hypothetical protein |
| cp29_00877 | cp18_01641 | cp22_00259 | hypothetical protein |
| cp29_00878 | cp18_01640 | cp22_00260 | hypothetical protein |
| cp29_00879 | cp18_01639 | cp22_00261 | hypothetical protein |
| cp29_00880 | cp18_01638 | cp22_00262 | hypothetical protein |
| cp29_00881 | cp18_01637 | cp22_00263 | hypothetical protein |
| cp29_00882 | cp18_01636 | cp22_00264 | hypothetical protein |
| cp29_00883 | cp18_01635 | cp22_00265 | hypothetical protein |
| cp29_00884 | cp18_01634 | cp22_00266 | hypothetical protein |
| cp29_00885 | cp18_01633 | cp22_00267 | hypothetical protein |
| cp29_00886 | cp18_01631 | cp22_00268 | hypothetical protein |
| cp29_00887 | cp18_01630 | cp22_00269 | hypothetical protein |
| cp29_00888 | cp18_01629 | cp22_00270 | hypothetical protein |
| cp29_00889 | cp18_01628 | cp22_00271 | hypothetical protein |
| cp29_00890 | cp18_01627 | cp22_00272 | hypothetical protein |
| cp29_00891 | cp18_01626 | cp22_00273 | hypothetical protein |
| cp29_00892 | cp18_01625 | cp22_00274 | hypothetical protein |
| cp29_00893 | cp18_01624 | cp22_00275 | hypothetical protein |
| cp29_00894 | cp18_01623 | cp22_00276 | hypothetical protein |
| cp29_00895 | cp18_01622 | cp22_00277 | hypothetical protein |
| cp29_00896 | cp18_01621 | cp22_00278 | hypothetical protein |
| cp29_00897 | cp18_01620 | cp22_00279 | hypothetical protein |
| cp29_00989 | cp18_01528 | cp22_00371 | hypothetical protein |
| cp29_01055 | cp18_01466 | cp22_00437 | hypothetical protein |
| cp29_01057 | cp18_01464 | cp22_00439 | hypothetical protein |
| cp29_01159 | cp18_01363 | cp22_00541 | hypothetical protein |
| cp29_01162 | cp18_01360 | cp22_00544 | Sulfate/thiosulfate import ATP-binding protein CysA |
| cp29_01216 | cp18_01309 | cp22_00598 | hypothetical protein |
| cp29_01219 | cp18_01306 | cp22_00601 | hypothetical protein |
| cp29_01225 | cp18_01300 | cp22_00607 | hypothetical protein |
| cp29_01245 | cp18_01281 | cp22_00627 | hypothetical protein |
| cp29_01246 | cp18_01280 | cp22_00628 | hypothetical protein |
| cp29_01247 | cp18_01279 | cp22_00629 | hypothetical protein |
| cp29_01248 | cp18_01278 | cp22_00630 | hypothetical protein |
| cp29_01250 | cp18_01276 | cp22_00965 | hypothetical protein |
| cp29_01251 | cp18_01275 | cp22_00964 | hypothetical protein |
| cp29_01252 | cp18_01274 | cp22_00963 | hypothetical protein |
| cp29_01253 | cp18_01273 | cp22_00962 | hypothetical protein |
| cp29_01254 | cp18_01272 | cp22_00961 | hypothetical protein |
| cp29_01292 | cp18_01235 | cp22_02490 | hypothetical protein |
| cp29_01353 | cp18_01174 | cp22_02429 | hypothetical protein |
| cp29_01490 | cp18_01038 | cp22_01301 | hypothetical protein |
| cp29_01501 | cp18_01027 | cp22_01290 | hypothetical protein |
| cp29_01518 | cp18_01009 | cp22_01273 | putative ABC transporter ATP-binding protein |
| cp29_01523 | cp18_01004 | cp22_01268 | hypothetical protein |
| cp29_01524 | cp18_01002 | cp22_01267 | Oligopeptide transport system permease protein OppB |
| cp29_01539 | cp18_00987 | cp22_01253 | hypothetical protein |
| cp29_01545 | cp18_00981 | cp22_01247 | hypothetical protein |
| cp29_01546 | cp18_00979 | cp22_01246 | hypothetical protein |
| cp29_01547 | cp18_00977 | cp22_01245 | hypothetical protein |
| cp29_01548 | cp18_00975 | cp22_01244 | hypothetical protein |
| cp29_01549 | cp18_00974 | cp22_01243 | hypothetical protein |
| cp29_01550 | cp18_00973 | cp22_01242 | hypothetical protein |
| cp29_01551 | cp18_00972 | cp22_01241 | hypothetical protein |
| cp29_01552 | cp18_00971 | cp22_01240 | hypothetical protein |
| cp29_01553 | cp18_00970 | cp22_01239 | hypothetical protein |
| cp29_01555 | cp18_00968 | cp22_01237 | hypothetical protein |
| cp29_01556 | cp18_00967 | cp22_01236 | hypothetical protein |
| cp29_01557 | cp18_00966 | cp22_01235 | hypothetical protein |
| cp29_01615 | cp18_00910 | cp22_01177 | hypothetical protein |
| cp29_01747 | cp18_00708 | cp22_02142 | hypothetical protein |
| cp29_01755 | cp18_00701 | cp22_02134 | hypothetical protein |
| cp29_01783 | cp18_00674 | cp22_02106 | hypothetical protein |
| cp29_01819 | cp18_00637 | cp22_02071 | Vitamin B12 import system permease protein BtuC |
| cp29_01835 | cp18_00622 | cp22_02055 | hypothetical protein |
| cp29_01889 | cp18_00567 | cp22_00966 | hypothetical protein |
| cp29_01958 | cp18_00500 | cp22_01993 | hypothetical protein |
| cp29_01959 | cp18_00499 | cp22_01992 | hypothetical protein |
| cp29_01961 | cp18_00497 | cp22_01990 | hypothetical protein |
| cp29_01970 | cp18_00488 | cp22_01981 | hypothetical protein |
| cp29_01972 | cp18_00486 | cp22_01979 | putative ABC transporter ATP-binding protein |
| cp29_01973 | cp18_00485 | cp22_01978 | putative ABC transporter ATP-binding protein |
| cp29_01987 | cp18_00471 | cp22_01964 | hypothetical protein |
| cp29_02116 | cp18_00340 | cp22_01835 | hypothetical protein |
| cp29_02119 | cp18_00338 | cp22_01832 | hypothetical protein |
| cp29_02123 | cp18_00334 | cp22_01828 | hypothetical protein |
| cp29_02172 | cp18_00285 | cp22_01779 | hypothetical protein |
| cp29_02193 | cp18_00266 | cp22_01758 | hypothetical protein |
| cp29_02194 | cp18_00265 | cp22_01037 | hypothetical protein |
| cp29_02202 | cp18_00257 | cp22_01029 | hypothetical protein |
| cp29_02225 | cp18_00234 | cp22_01006 | hypothetical protein |
| cp29_02236 | cp18_02127 | cp22_01412 | hypothetical protein |
| cp29_02238 | cp18_00223 | cp22_01414 | hypothetical protein |
| cp29_02239 | cp18_00222 | cp22_01415 | putative protein |
| cp29_02244 | cp18_00217 | cp22_01420 | hypothetical protein |
| cp29_02245 | cp18_00215 | cp22_01421 | hypothetical protein |
| cp29_02247 | cp18_00212 | cp22_01423 | hypothetical protein |
| cp29_02269 | cp18_00190 | cp22_01446 | hypothetical protein |
| cp29_02271 | cp18_00187 | cp22_01448 | hypothetical protein |
| cp29_02272 | cp18_00186 | cp22_01449 | hypothetical protein |
| cp29_02274 | cp18_00185 | cp22_01451 | hypothetical protein |
| cp29_02277 | cp18_00182 | cp22_01454 | hypothetical protein |
| cp29_02278 | cp18_00181 | cp22_01455 | hypothetical protein |
| cp29_02292 | cp18_00168 | cp22_01469 | hypothetical protein |
| cp29_02350 | cp18_00111 | cp22_01527 | hypothetical protein |
| cp29_02364 | cp18_00098 | cp22_01541 | hypothetical protein |
| cp29_02367 | cp18_00095 | cp22_01544 | hypothetical protein |
| cp29_02368 | cp18_00094 | cp22_01545 | hypothetical protein |
| cp29_02406 | cp18_00060 | cp22_01583 | hypothetical protein |
| cp29_02407 | cp18_00059 | cp22_01584 | hypothetical protein |
| cp29_02408 | cp18_00058 | cp22_01585 | hypothetical protein |
| cp29_02409 | cp18_00057 | cp22_01586 | hypothetical protein |
| cp29_02410 | cp18_00056 | cp22_01587 | hypothetical protein |
| cp29_02435 | cp18_00032 | cp22_01612 | hypothetical protein |
| cp29_02445 | cp18_00022 | cp22_01622 | Cryptic beta-glucoside bgl operon antiterminator |
| cp29_02446 | cp18_00021 | cp22_01623 | PTS system glucose-specific EIIA component |
| cp29_02447 | cp18_00020 | cp22_01624 | PTS system glucose-specific EIICBA component |
| cp29_02448 | cp18_00019 | cp22_01625 | hypothetical protein |
| cp29_02449 | cp18_00018 | cp22_01626 | hypothetical protein |
| cp29_02450 | cp18_00017 | cp22_01627 | hypothetical protein |
| cp29_02451 | cp18_00016 | cp22_01628 | hypothetical protein |
| cp29_02523 | cp18_02536 | cp22_01700 | hypothetical protein |
| cp29_02550 | cp18_02508 | cp22_01727 | hypothetical protein |
